# Towards an Understanding of the Mode of Action of Human Aromatase Activity for Azoles through Quantum Chemical Descriptors-Based Regression and Structure Activity Relationship Modeling Analysis

**DOI:** 10.3390/molecules25030739

**Published:** 2020-02-08

**Authors:** Chayawan Chayawan, Cosimo Toma, Emilio Benfenati, Ana Y. Caballero Alfonso

**Affiliations:** 1 Laboratory of Environmental Chemistry and Toxicology, Department of Environmental Health Sciences, Istituto di RicercheFarmacologiche “Mario Negri”—IRCCS, Via Mario Negri, 2, 20156 Milano, Italy; chayawan.chayawan@marionegri.it (C.C.); cosimo.toma@marionegri.it (C.T.); 2 Jozef Stefan International Postgraduate School, Jamovacesta 39, 1000 Ljubljana, Slovenia

**Keywords:** aromatase CYP19A1 enzyme, agonist, antagonist, quantum-mechanical descriptors, stereochemistry

## Abstract

Aromatase is an enzyme member of the cytochrome P450 superfamily coded by the CYP19A1 gene. Its main action is the conversion of androgens into estrogens, transforming androstenedione into estrone and testosterone into estradiol. This enzyme is present in several tissues and it has a key role in the maintenance of the balance of androgens and estrogens, and therefore in the regulation of the endocrine system. With regard to chemical safety and human health, azoles, which are used as agrochemicals and pharmaceuticals, are potential endocrine disruptors due to their agonist or antagonist interactions with the human aromatase enzyme. This theoretical study investigated the active agonist and antagonist properties of “chemical classes of azoles” to determine the relationships of azole interaction with CYP19A1, using stereochemical and electronic properties of the molecules through classification and multilinear regression (MLR) modeling. The antagonist activities for the same substituent on diazoles and triazoles vary with its chemical composition and its position and both heterocyclic systems require aromatic substituents. The triazoles require the spherical shape and diazoles have to be in proper proportion of the branching index and the number of ring systems for the inhibition. Considering the electronic aspects, triazole antagonist activity depends on the electrophilicity index that originates from interelectronic exchange interaction (*ω*_HF_) and the LUMO energy ($E_{\mathrm{LUMO}}^{\mathrm{PM}7}$), and the diazole antagonist activity originates from the penultimate orbital ($E_{\mathrm{HOMONL}}^{\mathrm{PM}7}$) of diazoles. The regression models for agonist activity show that it is opposed by the static charges but favored by the delocalized charges on the diazoles and thiazoles. This study proposes that the electron penetration of azoles toward heme group decides the binding behavior and stereochemistry requirement for antagonist activity against CYP19A1 enzyme.

## 1. Introduction

Azoles are compounds which have a wide range of applications such as antifungals, pesticides, and also as aromatase inhibitors for cancer patients. They are classified as mono-, di-, tri-, and tetrazoles based on the basis of the number of nitrogen atoms (1, 2, 3, or 4, respectively) present in the ring, and as thiazoles and oxazoles for rings containing N, S, and N, O atoms, respectively [1]. The nonbonded electrons of the heteroatom(s), especially nitrogen, enable the biological applications of these compounds against the cytochrome P450 enzymes. Cytochrome P450 enzymes have a heme prosthetic group, which promotes chemical reactivity through the formation of a dative bond with nonbonded electrons of azole chemical species, and hence interferes with natural biochemical reactions [2]. Relating to environmental and human health concerns, the prolonged use of these compounds as pesticides, antibacterials, and antifungals increases their concentration in air, soil, water, and living organisms. Moreover, the growing resistance of some microbes requires the discovery and development of new fungicides and antibacterial compounds [3,4]. 

The cytochrome P450 aromatase CYP19A1 enzyme is responsible for the main steps in the conversion of androstenedione into oestrogen during steroid genesis. Azole compounds have been shown to interfere with its biological catalysis and, as such, are referred to as endocrine disruptors [5]. Azoles are also classified as inactive, active agonist, and active antagonist with respect to their aromatase CYP19A1 activity, where active agonists and active antagonists both contribute to endocrine disruption. To distinguish the two activities, the antagonist is represented by the *p*IC_50_ (logarithmic inhibition concentration) and the agonist activity is represented by the *p*EC_50_ (logarithmic effective concentration). New antifungal and antibacterial azoles should be inactive against human cytochrome P450 aromatase CYP19A1 enzyme, while targeting specific CYP19A1 aromatase inhibitors. Thus, antagonists are required for oestrogen receptor positive postmenopausal breast cancer patients. Modeling of the inhibitors’ lone pair interactions with the iron of the heme group in the electronic and stereochemical environment of CYP19A1 is among the strategies for target specific drug design [6].

Recently, an X-ray characterized structure of CYP19A1 aromatase identified the van der Waals and polarizing regions in the reaction cavity to pucker the natural androgen substrate and catalyze the androstenedione to oestrogen biochemical aromatization reaction [7]. The hydrophobic amino acid residues are Arg115, Ile133, Phe134, Phe221, Trp224, Ala306, Thr310, Val370, Val373, Met374, and Leu477. The C3 and C17 atoms at different ends of androstenedione interact with Asp309 and Met374, respectively, through hydrogen bonding; this is the polar region of CYP19A1 [7]. The concerted aromatization reaction occurs at the interface of the van der Waals and polar regions at Ala306 and Thr310 to remove hydrogen at C2 (C2-H) using water as the catalyst. Asp309 helps to convert the ketone form to the alcohol in the presence of a proton. The rest of the process is catalyzed by the Fe^3+^ in the heme group in the presence of O_2_. The key interactions are hydrophobic, acidic, basic, and polar which have been exploited for the synthesis of steroidal aromatase inhibitors. However, most of these inhibitors have reduced activity due to irreversible inhibition. This is probably because of the strong polar interaction of the inhibitor (substituted at C6 of exemestane) with the Ser478 and Thr310 [5,8,9]. Reversible inhibition is one advantage of the azole compounds, although these strongly interfere with other cytochrome enzymes such as CYP2A6 and CYP3A4 [10]. 

To understand the aromatase inhibition (AI) action of the azoles, docking studies have been mostly employed [10,11,12]. A docking study by Suvannang et al., (2011) [11], found that Phe134, Trp224, Thr310, and Val373 amino acid residues have common hydrophobic interactions in all the nonsteroidal aromatase inhibitors (AIs), while other CYP19A1 enzyme–inhibitor interactions change with respect to the inhibitors’ shape, chemical constituents, and orientation of inhibitor in enzyme cavity. For example, comparing the docking of letrozole and anastrozole, anastrozole hydrogen bonds with Leu372, whereas, for letrozole, all hydrophobic interactions are only suggested. Another difference is that anastrozole interacts with Ser478 but letrozole interacts with Arg435. In addition to these differences, more than one pose for binding of azoles with aromatase is possible with only a small change of energy [10,11]. The docking experiments absolutely find the key functional groups and a functional group’s position in inhibitor for its activity; however underlying questions regarding driving the binding force and enzyme specific selectivity of inhibitors need to be answered. The quantum-mechanical descriptors encoding the information of electronic phenomenon are helpful to model and investigate hidden electronic interaction. Moreover, for global application of models, and the verification of the correctness of models, the docking experiments, fragment extraction with classification structural activity relationships, and regression models must be collaborated for enzymatic action modeling. However, a combination of two strategies, i.e., fragment extraction with advanced classification modeling and linear regression using quantum-mechanical descriptors, are being utilized for mechanistically interpretable modeling of mutagenicity data [13].

Two techniques are commonly used for the global application of the predictive modeling in toxicology, for example, employing either a large dataset using techniques such as machine learning methods or deriving mechanistically interpretable simple models [14,15]. Both techniques have been exploited to create predictive models for the antagonist activity of azoles with CYP19A1 [16,17,18,19,20]. Shoombuatong et al., 2018, reviewed this area and concluded that the modeling of nonsteroidal aromatase inhibition requires nitrogen-containing descriptors, polarizability, the energy of highest occupied molecular orbital (HOMO), the energy gap of highest occupied molecular orbital and lowest unoccupied molecular orbital (HOMO-LUMO gap), and descriptors for hydrogen bond acceptors [21]. A comparison of two multilinear regression QSARs for a dataset of diarylalkylimidazoles and diarylalkyltriazoles for AI, by Ghodsi et al., 2016 [22], Nagar et al., 2010 [23], and Ghodsi et al., 2016 [22], found that the Dragon descriptors, namely topological and van der Waals interactions, were significant, whereas Nagar et al., 2010 [23], modeled the binding interactions through molecular operating environment (MOE) based descriptors and found that van der Waals interactions relating, number of hydrogen bonds and bond angle potential energy were significant. These studies suggested that approximately the same information relating to the inhibition of the aromatase CYP19A1 is encoded by different descriptors for van der Waals and electrostatic interactions [22,23]. However, Ghodsi et al., 2016 [22], hypothesized the mode of action for the interaction between CYP19A1 and diarylalkylimidazole and diarylalkyltriazole molecules to be related to the shape and position of the isosurface of the HOMO orbitals. Nagar et al., 2010 [23], identified the different regions for hydrophobic, electrostatic, and steric interactions in azole compounds with CYP19A1 for inhibition using the CoFMA technique. It must be noted that QSAR models based on the diazole, triazole, and thiazole chemical classes have never been realized for the inhibition (antagonist) activity and, moreover, the agonist activity of azoles has not been reported for CYP19A1, as is indicated in our literature survey. The different robust QSAR models based on the chemical classes can be used to deduce the driving forces for antagonist or agonist activity by comparing the information of the descriptors in the different models. 

In this study, two main strategies, namely binary classification (agonist and antagonist activities) and regression modeling, were employed to model the agonist (*p*EC_50_) and antagonist (*p*IC_50_) properties of azoles on CYP19A1. The proposed classification strategy was utilized to extract structural alerts (SAs) for both agonism and antagonism from a subset of active azoles (listed in Table SA1) [24]. The behavior of the fragments extracted, with regard to activity, was correlated to descriptors obtained from MLR, and the dual mode of the action (agonistic/antagonistic) of azole, while interacting with CYP19A1, was explored. Therefore, combining classes of azoles, namely monazoles (thiazole/oxazole), diazoles (imidazole and benzimidazoles), and triazoles, having either agonist or antagonist activity, we attempted to extract SAs (fragments) and four classes of azoles, namely agonist monazoles (thiazole/oxazole), agonist diazoles (1,3-diazoles including imidazoles and benzimidazole), antagonist diazoles (1,3-diazoles, including imidazoles and benzimidazole), and antagonist triazoles (1,2,4-triazole) to be employed for regression analysis. The model building, validation methods, definition of descriptors, and statistical parameters are described in Section 3. 

## 2. Results and Discussion

### 2.1. Classification Modeling 

The classification model derived for two activity cases (agonist and antagonist) consisted of the structural alerts and fragments (see Table 1). A total of 11 SAs were extracted by employing the dataset described in Section 3 and Table SA1; four of these were associated with agonists and seven with antagonists. The SMARTS of the SAs, their structure, the associated activity, likelihood ratio (LR), accuracy, and other relevant information are reported in Table 1. The fragment with high (0.9 < acc < 1) and medium (0.8 < acc < 0.9) accuracy, and high LR values were considered as privileged SAs (for detail see Section 3.6). In addition, other statistical (accuracy) parameters were calculated for the classification problem starting from the numbers of true positive (TP), true negative (TN), false positive (FP), and false negative (FN) such as the real accuracy (Q_2_), the random accuracy (Q_2, rand_), and delta accuracy (∆Q_2 =_ Q_2_ − Q_2, rand_) of the complete model, and also for each fragment, as reported in Table SA2 of Supplementary Information. The real accuracy of the final classification model based on 11 SAs for all 78 compounds was 92.3%, the most probable random accuracy was 50.3%, giving the difference (delta accuracy ∆Q_2_) of 42% (see Section 3.6, and Table SA2 of Supplementary Information). This delta accuracy (maximum value = 50%) of 42% is considered as the real contribution of the model which was significantly above the most probable level of random accuracy, indicating a good quality classification model. 

The LR value was “inf” for most of the fragments; for the SA1 (monazole) and SA3 (triazole) fragments all compounds in these classes in our dataset were agonists or antagonist, respectively. This was regardless of the presence of other structural feature within the monazole and triazole classes contributing to opposing activity, although these classes have been classified with high accuracy for respective activities in literature studies [25,26,27,28,29,30,31]. An ideal value of accuracy was one, which means all predictions by the fragment or model were accurate (for detail see Section 3.6). The diazole class in our dataset was reasonably balanced for both activities (27 antagonist and 18 agonist), however, the 1,3-diazole derivative antagonists SA6 (benzylimidazole) and SA7 (1-phenyl-1*H*-imidazole) had a LR value of “inf”. The fragments, SA6 and SA7, were differentiated by the chain length as one carbon atom was present between the diazole ring and the benzene ring in SA6, whereas, in SA7, the diazole and benzene rings were directly connecting. The SA6 was more branched than SA7, however, the average activity of SA6 (average *p*IC_50_ = 9.79) was significantly greater than SA7 (average *p*IC_50_ = 7.87), thus, the branched benzyl group in SA6 imparted more inhibitory properties in diazoles to CYP19A1 than the phenyl group in SA7 (Table 1). It must be noted that both SA6 and SA7 fragments were present in only six diazoles (three for each).

The other antagonist fragments extracted were SA2 and SA9 with LR values “inf” and 11.3, respectively, and high accuracy (Acc) (Table 1). The fragments SA2 and SA9 were likely to be unique to provide the information, as these fragments were present in a large number of antagonists, including the molecules that belonged to triazole and diazoles classes (Table 1). However, fragment SA9 was associated with two incorrect predictions for diazole molecules, namely selumetinib and pifexole, where the pifexole contained only SA9 fragment and selumetinib contained agonist fragment SA8 along with SA9 (see Table 1 and the Supplementary Information Tables SA2 and SA1.8). There were only two common chemicals contained in the groups of compounds, i.e., (SA2, SA9) and (SA6, SA7), namely imazodan and liarazole, and two chemicals were common in SA2 and SA9 (see Table 1 and Supplementary Information Tables SA2, SA1.2, SA1.6, SA1.7, and SA1.9). Chemically, the SA2 and SA9 group is chlorobenzene and p-alkyl (ethyl) substituted chlorobenzene. However, for the diazoles, their contribution to inhibitory activity was not the same as for SA6 and SA7. The branched SA2 (p-alkyl substituted chlorobenzene) (average pIC_50_ = 8.40, more branched) imparted less inhibitory properties than SA9 (chlorobenzene) (pIC_50_ = 8.45, less branched) in diazoles, which was contrary to the behavior of SA6 (more branched) and SA7 (less branched). These observations showed that the position of branching in the diazoles has a role in the inhibition of diazoles in CYP19A1. This is in agreement with the observation by Ghodsi et al., 2016 [22], that an aromatic ring near the heterocyclic ring causes delocalization of HOMO electrons over the two rings and makes them less available to interact with the heme group and, as a consequence, SA7 (less branched) induced less inhibition as compared with SA6 (more branched) in diazoles. We concluded that the diazole molecules favor the inhibition of CYP19A1 by including the chlorobenzene fragment and not the branched p-alkyl (ethyl) substituted chlorobenzene. 

However, SA2 and SA9 fragments present in triazoles had the opposite effects with respect to branching increasing inhibition. All 15 antagonist molecules contained either SA2 or SA9 fragments and two molecules, ipconazole and fenbuconazole, contained both SA2 and SA9 fragments (see Table 1 and Supplementary Information Tables SA1.2 and SA1.9). The inhibitory properties of triazoles were more favored by the branched p-alkyl (ethyl) substituted chlorobenzene (average pIC_50_ = 7.89) than chlorobenzene (average pIC_50_ = 7.72) which was contrary to that in diazoles (Table 1). In conclusion, the inhibition properties changed with substitution of the same substituents in the different heterocyclic rings (diazole and triazole).

For the agonist activity, only two relevant fragments, SA4 and SA8, were identified. SA4 was a carboxylic functional group; the LR value was “inf” and Acc. high (0.93) (Table 1). The fragment SA8 was an amide functional group having a LR value of 4.96 and its statistical reliability was medium to high (0.81). Chemically, both groups offer conjugation over a short range which can cause the separation of electrostatic charges on the azole compounds. In conclusion, for the classical classification modeling, fragments in azoles having aromatic resonance (e.g., in benzene) were found to be antagonists, whereas small groups which separate the charges were found to control their agonistic activity. The fragments SA5, SA10, and SA11 had low LR, accuracy, and therefore poor reliability.

### 2.2. Regression Modeling

#### 2.2.1. Modeling of the Aromatase Antagonist Activity of Triazoles

The basis set employed for quantum-mechanical HF method was def2-SV(P), which employs split valence and polarization functions to atomic orbitals. The most robust and reliable QSAR model obtained is described in Equation (1),

(1) $$pIC50=1.4676+488.5007\omega_{\mathrm{HF}}+4.3535E_{\mathrm{PM}7}^{LUMO}+5.6602Eta\_shape\_Y$$

**Figure 1:**
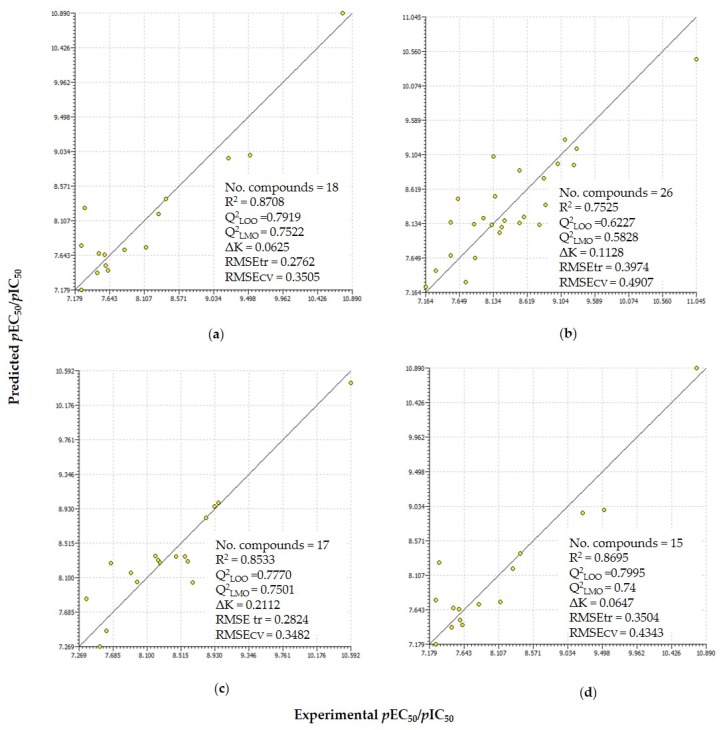
Scatter plots of the experimentally measured property and predicted activity obtained for four regression relations Equations (1), (2), (4), and (5) along with the statistical parameters for (**a**) agonist monazoles (thiazole/oxazole); (**b**) agonist diazoles (imidazoles and benzimidazole); (**c**) antagonist diazoles (imidazoles and benzimidazole); and (**d**) antagonist triazoles.

Equation (1) is dependent on three descriptors, namely electrophilicity index (ω), energy of lowest unoccupied molecular orbital (LUMO energy), and extended topological descriptor for shape of molecule (ETA shape index), which contribute positively to the aromatase antagonist activity. The *R*^2^ and *Q*^2^_LOO_ values were 0.87 and 0.79, respectively. The Q under influence of K (QUIK rule) (ΔK) value was 0.06, which suggested that the descriptors were not collinear (for detail see Section 3.5). The *Q*^2^_LMO_ value, 0.74, demonstrated the robustness of the model. Furthermore, the robustness of the model is visualized by the scatter plot (Figure 1). The standard residual errors in predictions and applicability domain of the model can be seen in the Williams plot (Supplementary Information Figure S4). Letrozole, which has the highest activity, lies at the threshold leverage value but it is well predicted. 

The descriptors influencing activity were electrophilicity calculated by the HF method. The electrophilicity index (ω) is the maximum flow of the electrons toward a molecule when it is immersed in an electron sea, which infers that triazole molecules were weakly attracting the electronic environment of the enzyme cavity. Interestingly, the interelectronic exchange interactions originated electrophilicity (ω_HF_) of the triazole molecules appeared in Equation (1) rather than interelectronic columbic interactions (ω_CORR_) or approximated full absolute electrophilicity (ω_PM7_ and ω_B3LYP_), despite all electrophilicity (ω_HF,_ ω_CORR,_ ω_PM7,_ and ω_B3LYP_) were employed for model building. The exchange interactions originate from the parallel spin of the electron, therefore, triazole molecules could also be interacting with the magnetic environment in the enzyme cavity due to the oxidation state of the iron (Fe^+3^ has odd electron) during inhibition. Conversely, chemical bond formation is predominantly initiated by the interelectronic columbic interactions [26,27]. In addition to the weak interactions, the strong noncovalent interactions also appear to be significant as indicated by the positive correlation of the LUMO with *p*IC_50_. During the chemical reactions or interactions, the LUMO was generally utilized to accept electrons, therefore, the LUMO of the triazoles was involved in π–π interactions, nucleophilic attack, and hard soft acid–base (HSAB) interactions. Therefore, the contour LUMO isosurface diagrams of the compounds were analyzed. These pictures depict that the contribution of the LUMO either lays on the phenyl and aromatic rings or atoms near the functional group (Supplementary Information Figure S2). For example, the letrozole and anastrazole (the most active compounds) contour maps of the LUMOs were laid over the phenyl rings. Conversely, for triademefon (least active compound), the LUMO contribution was near a nucleophilic site on the carbonyl functional group and for the israpafant (a compound having mean *p*IC_50_ and also the most structurally diverse triazole, see Supplementary Information Table SB2), it was on the thiazole aromatic ring (Supplementary Information Figure S2). Therefore, the π–π interactions originating due to the HSAB principle (LUMO of highest and medium active antagonists) have more impact than nucleophilic interactions (least active antagonist) on *p*IC_50_. It must be noted that the LUMO is a negative quantity but the positive sign of the LUMO coefficient (in terms of electron gain enthalpy it is negative, according to the Koopmans theorem) in Equation (1) indicates that the greater the electron accepting ability of triazoles, the greater the reversible inhibition for aromatase CYP19A1. 

The third descriptor (ETA) defines shape and gives the information about molecular bulk when an atom is connected to the three other non-hydrogen atom; consequently, it is a quantification of the tertiary atoms present in the molecules [28]. Mathematically, it can be defined as $\frac{\sum {\left(\alpha \right)}_{Y}}{\sum \alpha }$, where *α* is the vertex index for core count (size of atom) and *α*_Y_ is the core count of tertiary atoms in the extended topological scheme. The positive contribution of the shape index (tertiary carbon atom) to the antagonist activity revealed that compact molecules, with less surface area such as the spherical shape of antagonists, increase *p*IC_50_ value for enzyme CYP19A1 inhibition. These stereochemical aspects for triazoles are consistent with the QSAR study by Song et al., 2016 [17], which found that the descriptor “molecular volume” has a negative contribution to antagonist activity. 

#### 2.2.2. Modeling of Aromatase Antagonist Activity of Diazoles

The dataset for the diazole molecules contained 27 data points, however, trifumizole had to be excluded as it was observed to be both a structural and response outlier. The final dataset contained 26 molecules with imidazole and benzimidazole skeletons. The final model obtained from the genetic algorithm was:

(2) $$pIC50=3.5936-22.4173E_{\mathrm{PM}7}^{HOMONL}-103.9622ETA\_etaP\_B-0.6724NRS$$

The regression and linear QSAR parameters are presented in Table SB3. The *R*^2^ value is 0.75 and the cross-validated *Q*^2^_LOO_ is 0.62. The leave-many-out validation (*Q*^2^_LMO_) value for Equation (2) was 0.58. The triparametric equation was found to have independent variables as evident by the ΔK value of 0.11. The scattering of the activity points around the regression line and the graphical presentation of the residuals are reported in scatter and Williams plots in Figure 1 and Supplementary Information Figure S4. 

The ETA branching index ($\eta \prime_{B}$) is the non-hydrogen vertex count of the molecule and is calculated with respect to the reference alkane for estimation of branching in the molecule. Mathematically, it can be represented as Equation (3) [28]:

(3) $$\eta \prime_{B}=\frac{\eta_{N}^{local}-\eta_{R}^{local}+0.086\times N_{R}}{N_{v}}$$

where $\eta_{N}^{local}=1.414+\left(N_{v}-3\right)\times 0.05$, $\eta_{R}^{local}=\left\{\underset{i<j}{\sum }{\left(\gamma_{i}\gamma_{j}\right)}^{0.5}\right\}$, and *N*_R_ is the number of rings in the reference alkane, $\eta_{R}^{local}$ refers to the local composite index for the reference alkane, *Nv* refers to the number of non-hydrogen vertices, and γ*_i_* is the ratio of core count (α*i*) to valence electron count (*β*_i_) for *i*^th^ non-hydrogen vertex. Here, the symbol *η* should not be confused with quantum-mechanical hardness (Section 3.4). For aromatase CYP19A1 the branching index relative to the molecular size (ETA_etaP_B) was negatively correlated with the antagonist activity (Equation (2)) which means that branching was not favourable to antagonist activity. The other factor influencing the antagonist activity of diazoles was the number of ring systems (NRS). NRS was calculated as NRS = (E_t_ − E_r_) − (V_t_ − V_r_) + 1, where E_t_ and V_t_ represent the total number of bonds (edges) and atoms (vertices) in the whole molecule, respectively, and E_r_ and V_r_ are the total number of bond and atoms in rings present in the molecule [29]. 

Whilst being positively related to inhibition activity values (*p*IC_50_), in Equation (2), both the branching index (ETA_etaP_B) and the number of ring system (NRS) are negatively correlated to antagonist activity. These two descriptors refer to the stereochemistry of diazoles while interacting with CYP19A1 and their role has been explored as follows. For example, if we compare fadrozole (the highest active) and surinabant (the least active), fadrozole contains two NRS with branching index (0.0019) while surinabant has four ring systems (NRS = 4) with branching index (0.014). Surinabant has an alkyl side chain and substituents such as chlorine and bromine on all phenyl rings (Supplementary Information Figure S3 and Table SB3). On the other hand, the role of the branching index can be observed when NRS is the same (three) for liarozole (the highest active, branching index = 0.004) and zaldaride (the least active, branching index = 0.009), liarozole has only one tertiary carbon atom and one chlorine substituent on the phenyl ring, while zaldaride has two alkyl cyclic rings, inner, and alkyl side chains. Similarly, the NRS values of bificonazole and medetomidine are four and two, respectively. However, bificonazole (highest active compound with the highest NRS = 4) has the lowest branching index (−0.007) and medetomidine (the least active with least NRS = 2) has a high branching index (0.014). The structure of bificonazole has no substituents on phenyl rings, while medetomidine has an inner tertiary carbon and two methyl substitutions on the phenyl ring (see Supplementary Information Figure S3). Another case where the branching index of two molecules was nearly equal, for example, imazalil (more active, branching index = 0.01112, NRS = 2) and sertaconazole (less active, branching index = 0.01111, NRS = 3), the compound with the lower NRS was more active. Considering two compounds with approximately the same branching index and NRS (liarazole, branching index = 0.00474, NRS = 3 and imazodan, branching index = 0.00486, NRS = 3) they show similar stereochemistry properties but have different inhibitory activities indicating the role of electronic interactions between diazoles and aromatase enzyme. The structural alerts (SA6, SA7 and SA2, SA9) obtained from classical modeling suggested the role of branching in different positions for the stereochemistry aspect of inhibition. These results suggested the proportion of branching and numbers of aromatic ring systems in the diazoles were relevant factors for antagonist activity of diazoles. 

The descriptor $E_{\mathrm{PM}7}^{\mathrm{HOMONL}}$(energy of HOMO-1) refers to the second ionization enthalpy (Koopmans theorem) [26]. The energy of penultimate orbital ($E_{\mathrm{PM}7}^{\mathrm{HOMONL}}$) was negative for the diazoles and its negative coefficient in Equation (2) suggests that the tightly bound electrons at the second energy level of diazoles increase inhibition. A literature study by Nantasenamat et al., 2013 [14], also found that HOMO (calculated using higher level DFT) was negatively correlated to aromatase (CYP19A1) activity, however, for 1,2,3-triazole molecules. These observations showed that the electronic environment of the enzyme cavity interact with the HOMO, as well as HOMONL depending on the class of azole. The negative correlation between HOMONL and *p*IC_50_ for diazoles infers that the repulsive interelectronic interaction could play a role through the symmetry and energy of the diazole molecular orbital while interacting with the CYP19A1 for inhibition. Therefore, the HOMO, LUMO, and HOMONL contour graphs have been analyzed for the diazoles presented in Supplementary Information Figure S3. 

The HOMO contour graph of the diazole was concentrated on the 1,3 diazole ring alone (except sertaconazole). Conversely, the HOMONL contour graphs showed the isosurface was mainly occupied over aromatic system such as phenyl rings and other hetero aromatic ring (thiazole) and hetero atoms sulphur or nitrogen present in diazoles, as shown in Figure S2. However, the LUMO was located randomly on different groups of atoms in diazole molecules with respect to the HOMO contour graph, but it has the common overlapping regions with opposite sign of wavefunction in the contour graphs (LUMO and HOMONL) with respect to the HOMONL except for fadrozole, sertaconazole, 1-[2-(trifluoromethyl)phenyl]-1H-imidazole, timiperone, and zaldaride. With the exceptions of the overlapping of LUMO-HOMONL, the HOMONL was concentrated over a very small region (small fragment or one atom). For example, fadrozole, 1-[2-(trifluoromethyl)phenyl]-1*H*-imidazole, and timiperone have HOMONL contour graphs over one atom only and zaldaride, and sertaconazol have it over a small aromatic fragments. Interestingly, the docking calculations performed by Suvannang et al., 2011 [11], also showed that the nitrogen of fadrozole (HOMONL region) was near to the haem group of CYP19A1. These observations predict that the interactions between the haem group and HOMONL of the diazole are manifested either through the approach of electron densities of each other directly or indirectly through the LUMO.

#### 2.2.3. Electronic Interaction Aspects for Antagonist Activity of Diazoles and Triazoles

As discussed in Section 2.2.1 and Section 2.2.2, electronic interactions between inhibitor (triazoles and diazoles) and the aromatase enzyme CYP19A1 described through the positive coefficient of $E_{\mathrm{PM}7}^{\mathrm{LUMO}}$ and negative coefficient of $E_{\mathrm{PM}7}^{\mathrm{HOMONL}}$ (HOMO_PM7_-1) represented the flow of electrons toward inhibitors from the enzyme cavity. The inhibitors were interacting in the enzyme cavity having a negative potential environment with respect to the inhibitor electronic environment which depicts the role of electron withdrawing group (-R effect) in inhibitors. However, the manifestation of the azoles as an inhibitor for aromatase CYP19A1 has always been associated to coordinate (dative) interactions between the Fe^3+^ of haem group and the HOMO of the azole inhibitors [6,22]. It must be noted that the HOMO of triazole and diazole (triadimenol, fenbuconazole, triadimefon, and sertaconazol are exceptions) were laid over the 1,2,4-triazole and 1,3-diazole rings, respectively. In particular, for the molecules having the HOMO laid over the hetero ring (triazole, diazole), the polarizability of the hetero ring should be the determining factor for the coordinate bond length and, consequently, determine the stereochemistry involved and the noncovalent interactions between inhibitor and the enzyme’s amino acid residues. Kassimi et al. [30] showed that the 1,3-diazole ring is more polarizable than triazole rings (1,2,4- and 1,2,3-traizoles), therefore, the penetration of electrons for coordinate bond length in diazole should be more than in triazoles. The HOMONL of diazole molecules occupied over a small fragment or one atom only can be easily repelled by the negative potential of enzyme cavity through columbic forces and close proximity. However, the role of polarizability for dative bond formation and, as a consequence, the balance of the electrostatic environment through back bonding phenomena needed to be explored for the molecules having common regions in the HOMONL and LUMO contour graphs (see Supplementary Information Figure S3) [21,31]. Theoretically, back bonding and electrostatic balance phenomena have been studied for isolated haem groups with ligands [31], however, these have been overlooked for enzyme activities due to the computational complexity [30,31]. Conceptually, dative bonding and its resulting back bonding or electrostatic balance is a concerted process which is mediated through the HOMO and LUMO orbitals, respectively [31]. Among triazoles compounds, the 1,2,4-triazole ring has less polarizability (39.99 to 43.75 a^3^ units) [30], (region where the HOMO of the 1,2,4 triazoles resides, Figure S2) and it allows a lower flow of electrons toward the haem group, therefore, the electrostatic balance is maintained by the low energy LUMOs (−0.344 eV) to (−1.65 eV) of triazoles (see Supplementary Information Table SB2). For the diazoles, the 1,3-diazole ring has more polarizability (45.05 to 49.15 a^3^ units) [30] (region where the HOMO of 1,3-diazoles resides, Figure S2), therefore, it has greater penetrating electrons toward the haem group, which induces interactions with HOMONL to maintain the electrostatic balance in enzyme cavity. It must be noted that, the higher LUMO energy (−0.353 eV) to (−1.372 eV) (Supplementary Information Table SB3) of diazoles as compared with triazoles, and the common spatial region of the HOMONL and LUMO orbitals of diazoles, should be promoting factors for such interaction. In conclusion, the electronic interactions in the enzyme cavity were determining factors for the stereochemistry requirement of azoles for antagonist activity, which has also been reflected through the QSAR Equations (1) and (2) and classification modeling, where the spherical shape was required for triazoles and the proportion of number of ring system and branching index were determining factors for diazoles. 

#### 2.2.4. Modelling of Aromatase Agonist Activity for Diazole

To model diazole agonist activity, the dataset contained 18 molecules. Ataluren was excluded because it was identified to be a structural and response outlier in the initial QSAR modeling trials, thus, 17 molecules were modeled. The best descriptors-based model was:

(4) $$pEC50=8.6983-0.7252Qmax{\left(+\right)}_{PM7}-19.4372Eta\_dAlpha\_B+1.1130Eta\_betans\_d$$

*R*^2^, *Q*^2^_LOO_, *Q*^2^_LMO_, and ΔK were 0.85, 0.80, 0.78, and 0.21 respectively, as also presented in Figure 1. The scatter plot and Williams plot are depicted in Figure 1 and Figure S4, respectively. 

The triparametric Equation (4) contained two parameters, (*Q*max+_PM7_) and ETA_dAlpha_B, with negative coefficients for agonist activity. (*Q*max+_PM7_) represents the maximum positive charge holding non-hydrogen atom in diazole agonist molecules. ETA_dAlpha_B is calculated as 〈$\frac{{⎡\sum \alpha ⎤}_{R-\sum \alpha }}{N_{v}}$〉, where α is the core count, *α*_R_ is core count for the reference alkane, and N*v* is the number of non-hydrogen vertices, which measures the number of hydrogen bond acceptors [28]. Both negatively correlated terms represent the absolute localization of the charges (positive charges and negative charges), and therefore, these factors can be regarded as the limiting factors for agonist activity. The third parameter (Eta_βns_d) [28] refers to “number of lone pairs entering in the conjugation” which was positively related to activity and depicted that the delocalized partial negative charges in diazoles were increased the enzyme efficiency for natural substrate. The delocalized partial negative charge on the agonist diazole creates a more negative potential area around the enzyme than localized charges, and interacts with the polar regions in CYP19A1 enzyme to enhance the enzymatic action [7]. The electrostatic forces generated by *Q*max+_PM7_ and Eta_dAlpha_B are strong forces which have more tendency to form strong bonds with opposite charge than delocalized charges, therefore, they do not favour agonist activity. 

#### 2.2.5. Modeling of Agonist Activity for Thiazole/Oxazole

The thiazole/oxazole compounds contain a sulphur/oxygen atom along with the nitrogen atom in the azole ring. There are 18 compounds and most of the compounds contain the amino (-NH_2_) and alkyl ether (-OR) groups. The following (5) was obtained:

(5) pEC50=7.3704−8.0767GGI9+0.1177F03(C−C)+0.5873F04(N−O)

*R*^2^_,_ *Q*^2^_LOO_, and *Q*^2^_LMO_ were 0.87, 0.79, and 0.74, respectively, for Equation (5). All three descriptors were topological, GGI9 was negatively correlated, whereas F03(C-C) and F04 (N-O) were positively correlated with activity. The descriptor F04 (N-O) is the “frequency of occurrence of nitrogen and oxygen (N and O) after four connected atomic positions” and similarly F03(C-C) is the “frequency of occurrence of carbon after every three connected atomic positions” [39]. 

F04[N-O] suggests that nitrogen (N) and oxygen (O) should be appropriately distant from each other, which probably enables the lone pair of electrons to be conjugated (delocalisation of charge). F03[C-C] represents the abundance of the carbons in the molecules and controlling factor for hydrophobicity/hydrophilicity and channels for the electron delocalisation of electrons. 

The charge transfer index has been found to correlate with the dipole moment which could represent the presence of the absolute charges on atoms the molecule [40]. The long order charge transfer, namely GGI9 was negatively correlated with agonist activity. Comparing with diazole agonists, the maximum positive charge on the atom was negatively correlated. The QSAR models for the thiazole and diazole agonists have different descriptors, however, Equations (4) and (5) reflected similar information through different descriptors. For the agonist activity, the delocalization of the electrons and formation of mild absolute charges during delocalization of agonist molecule were the main characteristics. 

## 3. Materials and Methods

### 3.1. Data Collection

The starting dataset was collected from the Tox21 library considering only Tox21_Aromatase_Inhibition (activity test). This contained 20,992 compounds encoded as SMILES, name, and CAS number [41]. The assay was performed using aromatase breast cancer cell line (MCF-7 aro) (cell-based assay) and the concentrations of testosterone (an androgen and estradiol (an oestrogen)) were measured before and after exposure to azole compounds tested. The qualitative outcome was recorded as active agonist, active antagonist, and inactive, where quantitative agonist and antagonist activities were expressed in nanomolar (nM) units represented by AC_50_ in the original database [41]. 

### 3.2. Data Curation

The curation procedure of the data involved the retrieval of SMILES following the workflow developed by Gadaleta et al., 2018 [42]. The maximum purity was labelled “A” and only compounds with this label were considered. The detection of inorganic compounds, organometallic compounds, mixtures, neutralization of salts, tautomeric forms, and chemotype normalization were performed using the KNIME platform [43]. The compounds with inconclusive assay outcomes were discarded and duplicate structures were classified into two cases as follows: (i) activity range lower or equal to 1:3, and (ii) activity range higher than 1:3. In the first case, the mean of the activity was calculated, and in the second case, the structures were rejected. There were 3459 compounds that were kept from the original dataset which had the purity “A” label. Furthermore, 67 compounds with ambiguous values, 10 compounds with trace element or inorganic compounds, 3 mixtures, 6 duplicates, and 6 ionic liquid compounds were removed. After this, the dataset was subjected to a manual inspection process and 119 compounds were found to have incorrect structures, and therefore removed. At this point, the dataset was comprised of 3248 compounds, and was filtered to extract azoles only. The total number of azoles was 351 and the distribution of compounds in the dataset considering the numbers of nitrogen in the azole ring. Activity is shown in Supplementary Information, Tables SB2–SB5. The quantitative outcome in nanomolar (nM) units was converted to molar (mole/litre) using the formulae (−logAC_50_ + 9). 

### 3.3. Dataset

The 351 azoles contained substances with different numbers of nitrogen in the heterocyclic ring. There were 82 monoazoles (including oxazoles and thiazoles) present out of which 61 were inactive compounds and 21 were active compounds (18 agonist and 3 antagonist). For the diazoles, the total was 198 out of which 151 were inactive and 45 were active compounds (18 agonist and 27 antagonist). Similarly, for triazoles, the total was 47 which contained 26 inactive and 21 active compounds (6 agonist and 15 antagonist). Furthermore, 3 antagonist monoazoles and 6 agonist triazoles were removed from the curated dataset because there were too few for modeling purposes. Finally, through the multilinear regression analysis, four chemical sets, namely 15 antagonist triazoles, 27 antagonist diazoles, 18 agonist diazoles, and 18 agonist thiazoles were employed to derive four regression models. In addition, union of agonist and antagonist molecules (agonist ∪ antagonist) of this set was employed to extract the SAs, where antagonist and agonist was codified as 0 and 1, respectively (see Supplementary Information Tables SB2–SB5 and SA1).

To explore the diversity of the chemical space, the Tanimoto index [44] employing PubChem fingerprints was used as similarity measure. The distance matrix was computed using KNIME [43] and visualized through heat maps for the final full active dataset and classes of azoles, as stated above for MLR modeling. The heat maps are shown in Figure S1a (Supplementary Information Figure S1a,b) for full active dataset and classes of azoles, respectively, where green points indicate the highest similarity between two chemicals and blue points correlate to lower similarity. 

### 3.4. Descriptor Calculation

For the quantum-mechanical descriptor calculations, the SMILES of the chemicals were transformed to three-dimensional (3D) structures using MarvinSketch 18.10.0 and molecular coordinates were retrieved [45]. These coordinates were employed for the structure optimization using parametric model (PM7) semi-empirical method on Gabedit platform [46,47,48]. To find the true minima of every geometry, optimizations were repeated until no imaginary frequency was found in frequency calculations at the same level of theory. The geometry obtained using the PM7 method was further employed for single point calculations at Hartree–Fock (HF) [49] and Beke three parameters Lee Yang Parr (B3LYP) [50,51] method using def2-SV (for antagonist triazole) and def2-TZVP (for antagonist diazoles for heavy sulfur atom) basis sets [52] with the ORCA 3.0.3 program [53]. The semi-empirical PM7 and B3LYP methods take care of the dynamic electron correlation through the empirical parameters and exchange correlation functional, respectively, while the HF ignores the dynamic electron correlation but accurately estimates the exchange interaction within the complete basis set limit. The HF calculated descriptor is helpful to deduce the spin originated interactions originating in the enzyme substrate (Fe^3+^, have unpaired of electron) and ligand molecules, during binding. Additionally, the PM7 and B3LYP derived descriptors were calculated and employed for models to compare their descriptors performance, and therefore have a robust and economical model. The calculated quantum chemical descriptors include the energy of the molecule ($E_{\mathrm{QM}}$), energy of the highest molecular orbital energy ($E_{\mathrm{QM}}^{\mathrm{HOMO}}$), energy of the lowest unoccupied molecular energy ($E_{\mathrm{QM}}^{\mathrm{LUMO}}$), energy of the next level to the HOMO and LUMO ($E_{QM}^{\mathrm{HOMONL}/\mathrm{HOMO}-1}$ and $E_{QM}^{\mathrm{LUMONL}/\mathrm{LUMO}-1}$), and density functional descriptors [54], namely electronegativity ($\chi_{\mathrm{QM}}$ *=* $\left[\frac{\left(-E_{\mathrm{QM}}^{\mathrm{HOMO}}\right)+\left(-E_{\mathrm{QM}}^{\mathrm{LUMO}}\right)}{2}\right]$), hardness ($\eta_{QM}=\left(E_{\mathrm{QM}}^{\mathrm{LUMO}}-E_{\mathrm{QM}}^{\mathrm{HOMO}}\right)$, and electrophilicity index ($\omega_{\mathrm{QM}}=\frac{\chi_{\mathrm{QM}}^{2}}{2\eta_{\mathrm{QM}}}$), where subscript (QM) refers to the quantum-mechanical method (HF, B3LYP, and PM7) employed. To calculate electron correlation based descriptors (*D*_CORR_ = D_B3LYP_ − D_HF_), the frontier orbitals obtained in single point calculations at the HF and B3LYP levels were employed [55]. The electrostatic charges on azole molecules were extracted from the optimized geometry of PM7 output files. Besides these, various 2D descriptors have been used encompassing, extended topological atoms (ETA) indices, constitutional indices, ring descriptors, connectivity indices, functional group counts, atom centered fragments, atom type E-state, 2D- atom pairs, and molecular properties computed from PaDEL-Descriptor and Dragon software tools [52,56], in order to explore the lipophilicity and steric aspects of the interactions of molecules with the enzyme. All computed descriptors are listed in Supplementary Information Tables SB2–SB5. 

### 3.5. Regression Model Development and Validation

The linear regression method implemented within the QSARINS toolbox [57] was employed to model the experimental *p*IC_50_ and *p*EC_50_ using the calculated descriptors. A genetic algorithm was applied for variable selection for each model to obtain the optimal combination of descriptors and their regression parameters. For the GA, the population size was set to 10,000 generations, crossover and mutation rate were established at 0.8 and 0.2, respectively, with leave-one-out (*Q*^2^_LOO_) as the fitness function. Furthermore, the Williams plot, which is a plot between the standardized residual versus leverage (*h*), was analyzed to detect the structure and response outliers for all models. The compounds having standardized residuals of more than 3.0 standard deviation units were considered as response outliers and compounds with leverage greater than warning leverage h* (*h* > h*) were considered structural outliers. The robustness of all four models developed was checked using different metrics including determination coefficient (*R*^2^) (optimally greater than 0.7) and cross-validated (CV) *R*^2^ (*Q*^2^_LOO_ or *Q*^2^_CV_) using leave-one-out (LOO) method (optimally, greater than 0.55). The mean absolute error (*MAE*) and root mean square error (*RMSE*) for training and cross-validation runs were analyzed. Furthermore, model validation was performed using cross-validated leave-many-out (*R*^2^) (*Q*^2^_LMO_) taking 30% of the compounds in the prediction set in 2000 iterations (values optimally greater than 0.50) and Y scrambling (*Q*^2^_Yscr_) procedures. The Y scrambling procedure was employed by rearranging the activity values among all the compounds keeping their descriptor values the same and models were built in 2000 iterations. The performance of scrambling was measured by *Q*^2^_Yscr_ which must be as low as possible from the actual *Q*^2^_LOO_ value. The descriptor collinearity was verified using the QUIK rule (Q under influence of K) [58] with ΔK greater than 0.05. The QUIK rule is defined by the delta K (ΔK) which is the difference between K correlation of descriptors and activity (K_xy_) and K correlation between the descriptors (K_xx_). A greater value of ΔK represents less collinearity between the descriptors in model. The threshold value of ΔK is 0.05 [50]. The minimum ratio between the number of compounds and the number of the descriptors was five.

### 3.6. Structure Activity Relationship Modeling and Evaluation

To build the classification model, the SARpy software (version 1.0) was used. SARpy is a knowledge extractor tool to obtain relevant substructures and generate new knowledge analyzing a dataset of binary activity classes [24]. The precision was defined as “min” in order to obtain robust structural alerts. A substructure filter was applied to identify the compounds containing the extracted fragments and their distribution within the classes, using RDKit node of KNIME [43] (see Supporting Information Tables SA1.1 and SA1.11). The performance measuring parameter for each structural alert, the accuracy (Acc) (Equation (6) and likelihood ratio (Equation (7)) value were calculated through predictions made by fragments using the KNIME platform and SARpy software (cross-validation), respectively [24,59]. In the case of Equation (7), it is important to highlight that the format of the right member depends on the type of fragment under consideration, for example if the fragment corresponds to an agonist SA, Equation (7) remain with the same format as presented below, but if the fragment is an antagonist SA, the multiplication factor on the right is inverted (i.e., Total Agonist/Total Antagonist). In the cases where more than one SA was identified within a structure, the prediction was based on the SA with the highest LR value.

(6) $$\mathrm{Accuracy}\;\left(\mathrm{Acc}\right)=\frac{\mathrm{Total}\;\mathrm{number}\;\mathrm{of}\;\mathrm{True}\;\mathrm{prediction}\;\mathrm{by}\;\mathrm{SA}}{\mathrm{Total}\;\mathrm{number}\;\mathrm{of}\;\mathrm{prediction}\;\mathrm{by}\;\mathrm{SA}}$$

(7) $$\mathrm{LR}=\frac{\mathrm{True}\;\mathrm{Prediction}\;\mathrm{by}\;\mathrm{SA}\;}{\mathrm{Wrong}\;\mathrm{Prediction}\;\mathrm{by}\;\mathrm{SA}}\times \frac{\mathrm{Total}\;\mathrm{Antagonists}\;}{\mathrm{Total}\;\mathrm{Agonists}\;}$$

The accuracy calculated using Equation (6) refers to the ability of SA to yield the correct predictions. The accuracy can take values in a range of 0–1, in this sense, values close to one were desired, and were interpretated as a better performance during the classification [59]. All predictions by certain fragment are true if Acc = 1 and LR = infinite (inf). However, incorrect predictions are influenced by the accuracy of the given SA, the presence of other SAs with a similar accuracy value for opposite cases and also by the stereo-chemical environment of the SAs in the azole compounds. Therefore, levels of accuracy (Acc), low (Acc ≤ 0.6), low to medium (0.6 < Acc ≤ 0.7), medium (0.7 < Acc ≤ 0.8), and high (0.9 < Acc ≤ 1) were assigned to each SA. The assignation of qualitative levels of accuracy facilitated the interpretation of results and the evaluation and levels of accuracy (Acc) of SAs were also compared with the literature with respect to their consistency. The accuracies of SAs calculated using Equation (6) were found to be similar when cross checked with literature studies [25,26,27,28,29,30,31] (see Table 1). The LR values calculated using Equation (7) gave a measure of the degree of accurate predictions using the distribution of the SA between the activities (agonist and antagonist compounds) and the ratio of true and wrong predictions. The high LR values mean that a SA was predominantly found to contribute to one of the two activities, and the value "inf" means the alert was a perfect classifier. The value “inf” is the ideal value of LR, which means that the number of wrong predictions was zero, and accordingly, the division by zero (the denominator of Equation (7)) tends to infinity. The largest values of LR are interpreted as the highest relevance of the SA; however, unlike the accuracy where the numerical range is well defined, the wide numerical range of LR values could be difficult for interpretation. Additionally, the real accuracy, the random accuracy, and the difference between these two parameters, for the classification model and for each SA, were calculated using the methodology proposed by Lučić et al., 2019 [60], and Batista, et al., 2016 [61]. The outcome is presented in the Supplementary Information Table SA2. The difference of the real accuracy of SA and the random accuracy is called delta accuracy which can have an ideal value of 0.5. The structural alert with a high value of delta accuracy is regarded as privileged structural fragments (SAs).

## 4. Conclusions

Human aromatase activity has been studied for azole classes comprising of triazoles, diazoles, and thiazoles for their reversible inhibition and agonist activity. The classification modeling suggested that the chemical nature and position of substituents (chemical groups) on diazoles and triazole ring had different contributions to inhibition, while functional groups having resonating charges have a significant role for agonist activity. The regression model of triazoles for antagonist activity suggested that the electrophilicity originated from the interelectronic exchange interaction (*ω*_HF_), the LUMO energy and spherical shape were the key factors. The antagonist activity of diazoles was electronically a function of HOMONL energy and stereochemically a function of branching index and number of ring system (NRS). The literature studies [21,30,31], contour map of frontier orbitals (Figures S2 and S3), and regression Equations (1) and (2) suggested the significance of electrostatic balance/back bonding during dative interactions between enzyme CYP19A1 and azole molecules should be incorporated with the appropriate stereochemistry of azoles for effective inhibition or vice versa. Regression Equations (4) and (5) showed that localized charges have a negative contribution to the agonist activity, whereas the delocalized charges in diazoles and thiazoles increase the agonist behaviour with respect to the CYP19A1 aromatase enzyme. Interestingly, such mechanistic explanatory QSARs for CYP19A1 inhibition have never been reported in the literature and, moreover, agonist activity has not been the subject of published QSAR models, despite its importance to identify potential endocrine disruptors through the CYP19A1 enzyme.

## Figures and Tables

**Table 1:** Structural fragments for agonist and antagonist activity on CYP19A1 obtained from classification modeling along with their statistical parameters.

SA_ID	Name	SMARTS ^1^	Structure	Activity Related	LR Value Using Eq. 7	Accuracy (Acc) of SA Using Eq. 6	Statistical Reliability	Literature Reliability [17,32,33,34,35,36,37,38]	Relevant Information of Selected SA and Distributions
SA1	1,3-thiazoles	c2cscn2	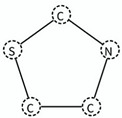	Active agonist	inf	1.0	High 0.9 < Acc ≤ 1	Medium	-
SA2	para substituted chlorobenzenes	Clc1ccc(CC)cc1	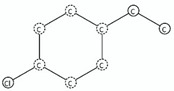	Active antagonist	inf	1.0	High 0.9 < Acc ≤ 1	High	Antagonist Diazoles (*n* = 9; Average *p*IC_50_ = 8.40) Antagonist Triazole (*n* = 7; Average pIC_50_ = 7.89)
SA3	1,2,4-triazoles	n1cncn1	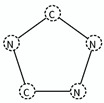	Active antagonist	inf	1.0	High 0.9 < Acc ≤ 1	High	-
SA4	carboxylic acids	C(=O)O	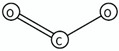	Active agonist	12.83	0.92	High 0.9 < Acc ≤ 1	High	Agonist Diazoles (*n* = 7; Average*p*EC_50_ = 8.88) Agonist Monozoles(*n* = 4; Average*p*EC_50_ = 9.09; Incorrect prediction = 1 triazoles)
SA5	ethyl(propyl)amine	CCCN(CC)	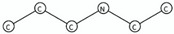	Active antagonist	1.71	0.67	Low to medium 0.6 < Acc ≤ 0.7	Low	-
SA6	benzylimidazoles	c1cn(cn1)C(c1ccccc1)	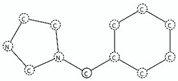	Active antagonist	inf	1.0	High 0.9 < Acc ≤ 1	High	Antagonist Diazoles (*n* = 3; Average *p*IC_50_=9.79)
SA7	1-phenyl-1H-Imidazoles	c1c(cccc1)n1ccnc1	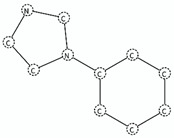	Active antagonist	inf	1.0	High 0.9 < Acc ≤ 1	High	Antagonist Diazoles (*n* = 3; Average *p*IC_50_ = 7.87)
SA8	Formamides	C(=O)N	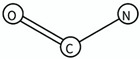	Active agonist	4.96	0.81	Medium to high 0.8 < Acc ≤ 0.9	Medium	Agonist Diazoles (*n* = 9; Average pEC_50_ = 8.53) Agonist Monozoles (*n* = 8; Average*p*EC_50_ = 8.47; Incorrect prediction = 4 diazoles)
SA9	chlorobenzenes	Clc1cccc(c1)	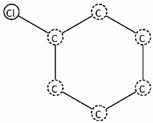	Active antagonist	11.57	0.93	High 0.9 < Acc ≤ 1	High	Antagonist Diazoles (*n* = 16; Average *p*IC_50_ = 8.45) Antagonist Triazoles (*n* = 11; Average *p*IC_50_ = 7.72; Incorrect prediction = 2 diazoles)
SA10	Carbon chain	CC	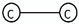	Active antagonist	1.67	0.66	Low to medium 0.6 < Acc ≤ 0.7	Low	-
SA11	purines	c1ncnc2c1ncn2	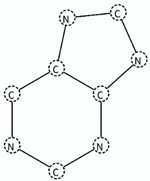	Active agonist	2.33	0.67	Low to medium 0.6 < Acc ≤ 0.7	Low to medium	-

^1^ SMARTS structures obtained from SMARTSanalyzer, Analyze Chemical Patterns, https://smartsview.zbh.uni-hamburg.de/. The discontinues circles represent the aromatic atoms and the continues circles the aliphatic atoms.

## Supplementary Materials

The following are available online, Excel file SA SA1: Aromatase database used for classification modeling, SA2: Output of the classification modeling, SA1.1–SA1.11: Compounds containg each fragment and the description of the fragment. Excel file SB: SB1: Full curated dataset, SB2–SB5: Activity data of compounds extracted from SB1 along with their calculated descriptors, Supporting Figures: Heat maps, quantum mechanical interactions. Figure S1: The heat maps showing the diversity of the data set; Figure S2: Representations of HOMO and LUMO isosurfaces for the antagonist triazoles; Figure S3: Representation of HOMO-1 (HOMONL), HOMO and LUMO isosurfaces of antagonist diazoles; Figure S4: Williams plots of leverage vs standardised residuals for equations (1,2,4,5).
