# Supplementary material for: Towards an Understanding of the Mode of Action of Human Aromatase Activity for Azoles through Quantum Chemical Descriptors-Based Regression and Structure Activity Relationship Modeling Analysis
Source: Molecules. 2020 Feb 8;25(3):739. doi: 10.3390/molecules25030739 (PMC7037385; doi:10.3390/molecules25030739)
Supplement: Supplementary file 1 [file molecules-25-00739-s001.zip › molecules-687501-supplymentary-final/molecules-687501-figures.pdf]

Supplementary Materials

Figure S1(a)

Full Data set

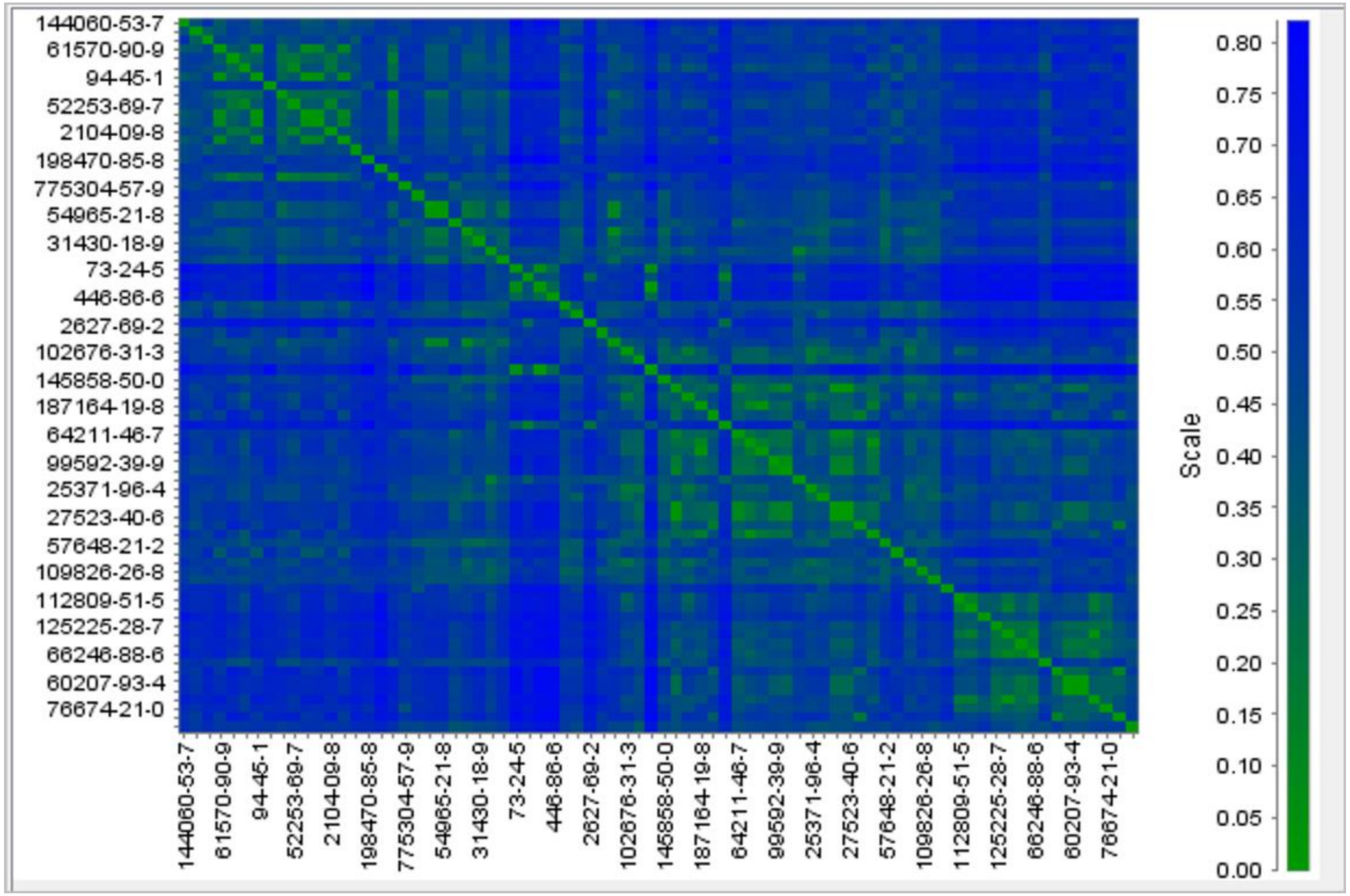

Figure S1(b)

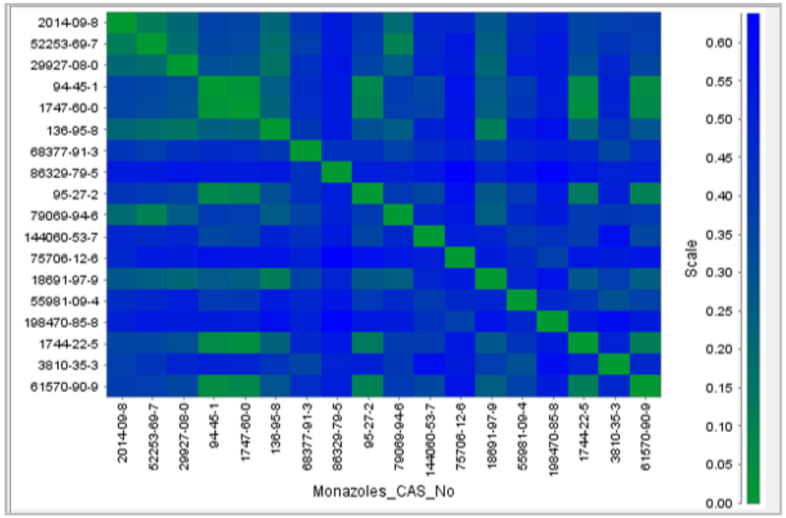

(c)

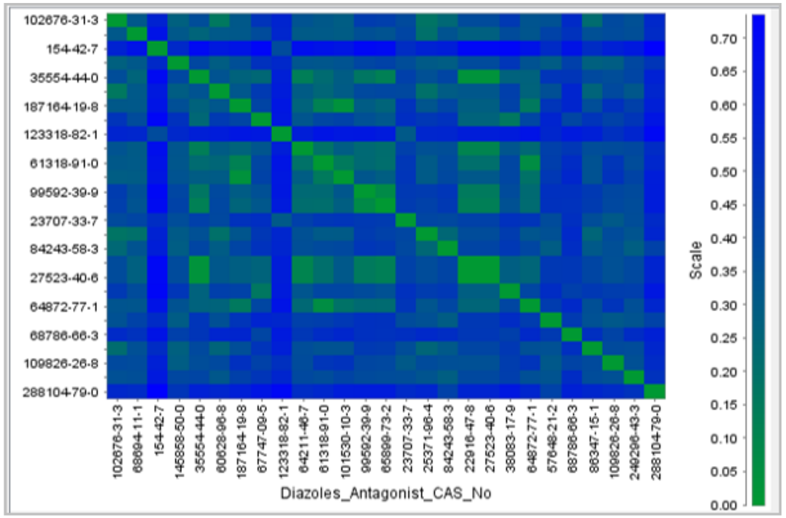

(d)

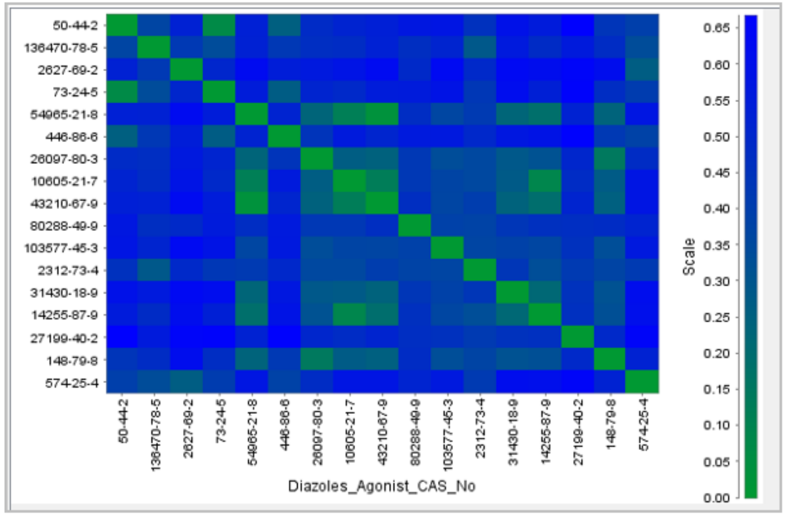

(e)

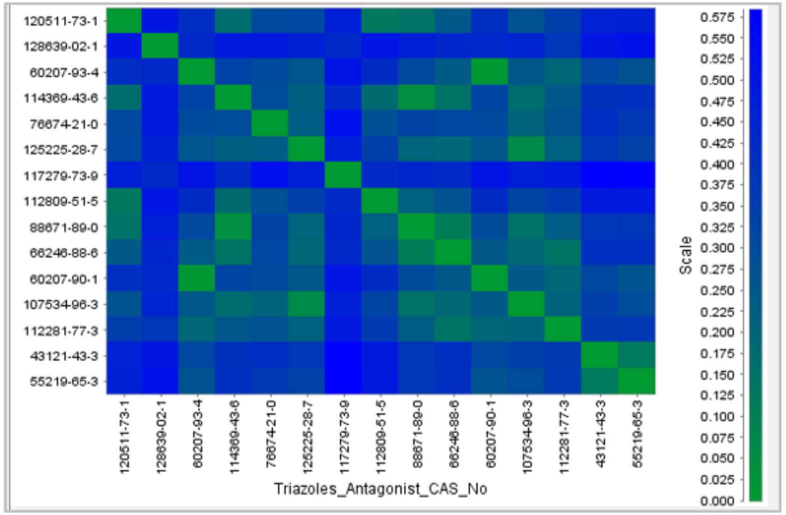

(f)

Figure S1: The heat maps showing the diversity of the data set. a.) Heat map for full data set. b) Heat map for the classified data set based on activity and class of the azole. On left side agonist monazoles (Thiazole/oxazole) (c) and agonist diazoles (imidazoles and benzimidazole) (e). On right side antagonist diazoles (imidazoles and benzimidazole) (d) and antagonist triazoles (f).

**Molecule**	**HOMO**	**LUMO**
Litrazole	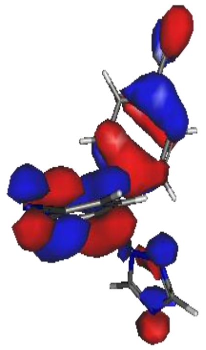	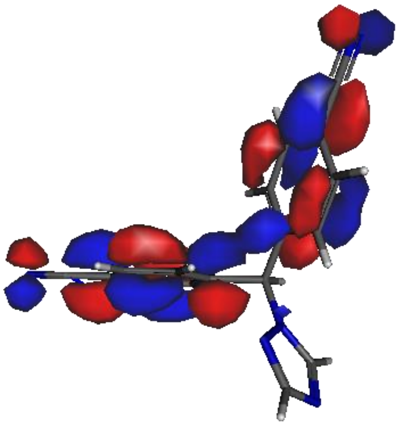
Anastrazole	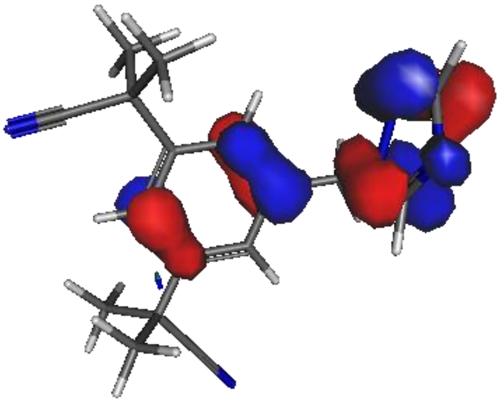	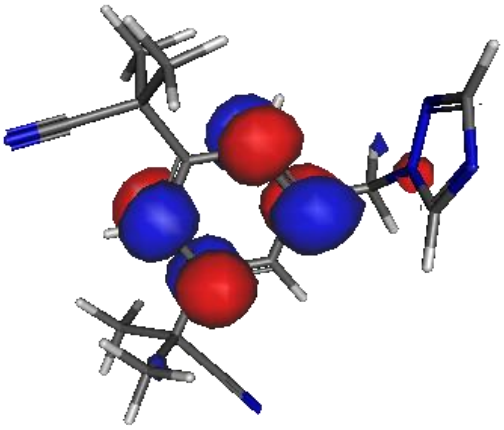
Flusilazole	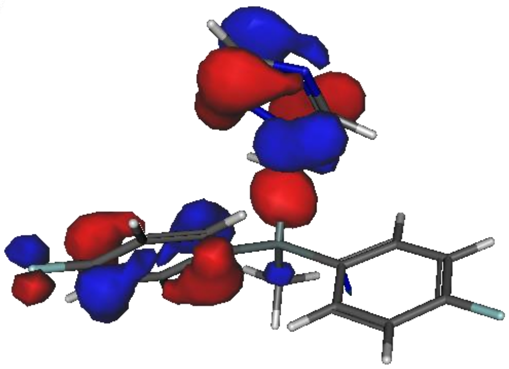	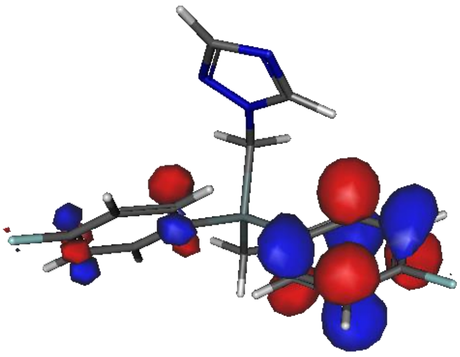
Ipiconazole	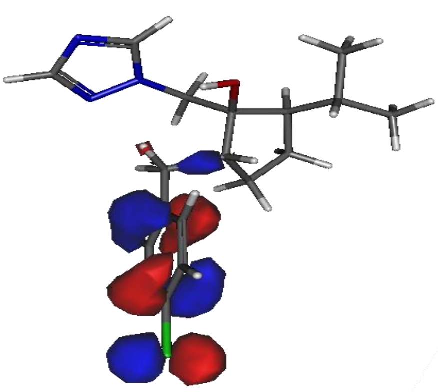	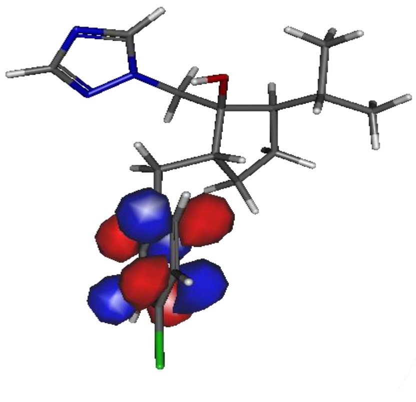

Myclobutanil

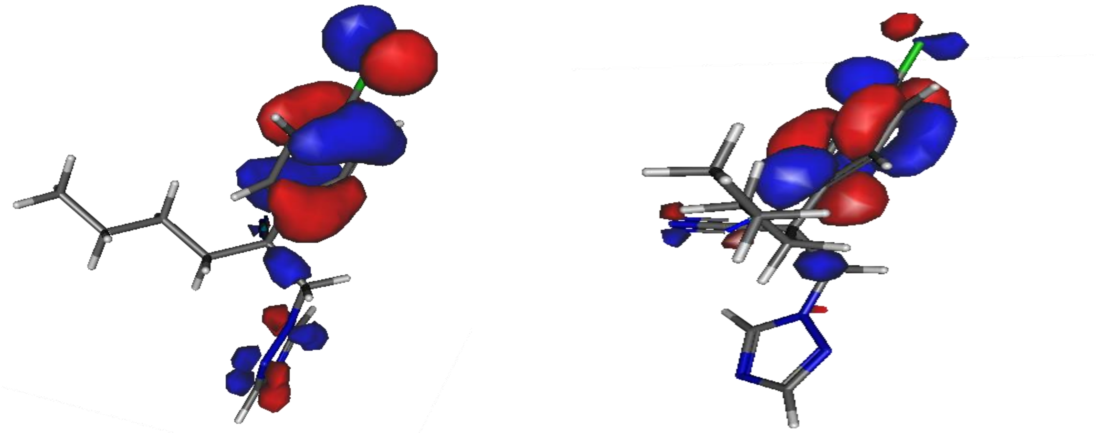

Tetraconazole

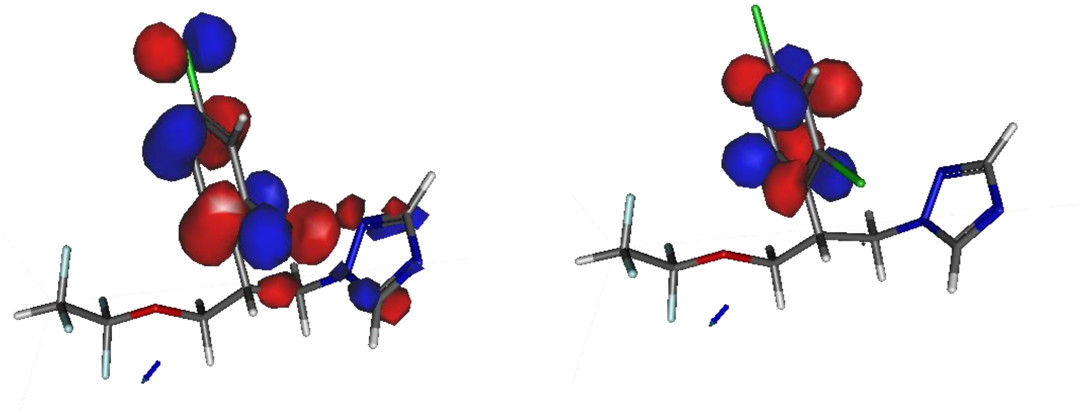

Penconazole

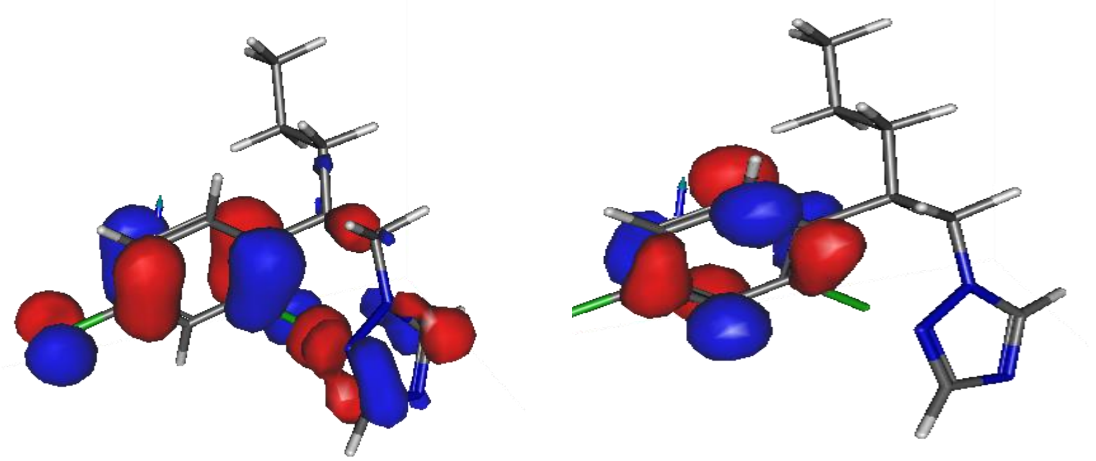

Israpafant

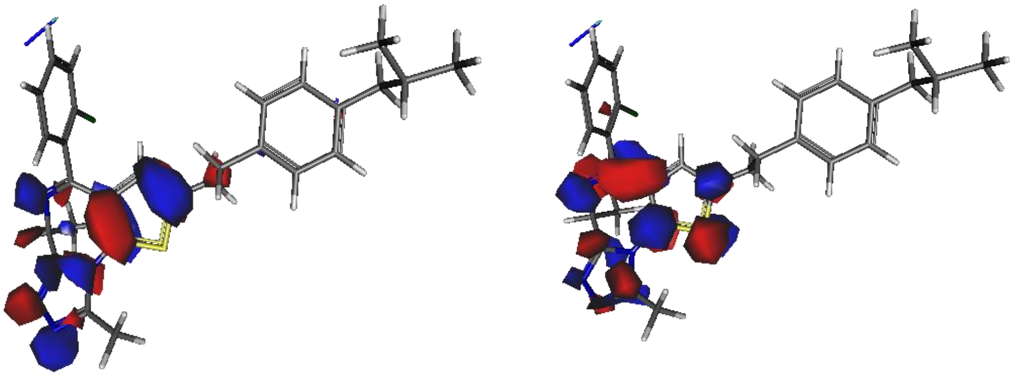

Triadimenol

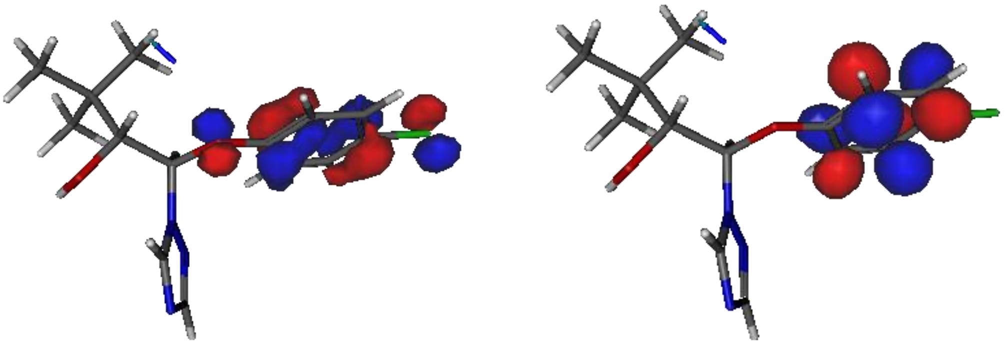

Etaconazole

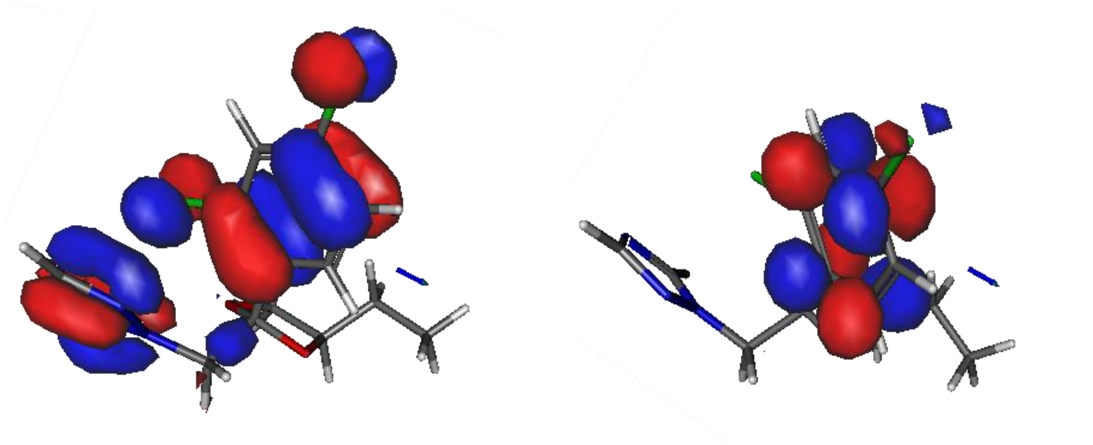

Propiconazole

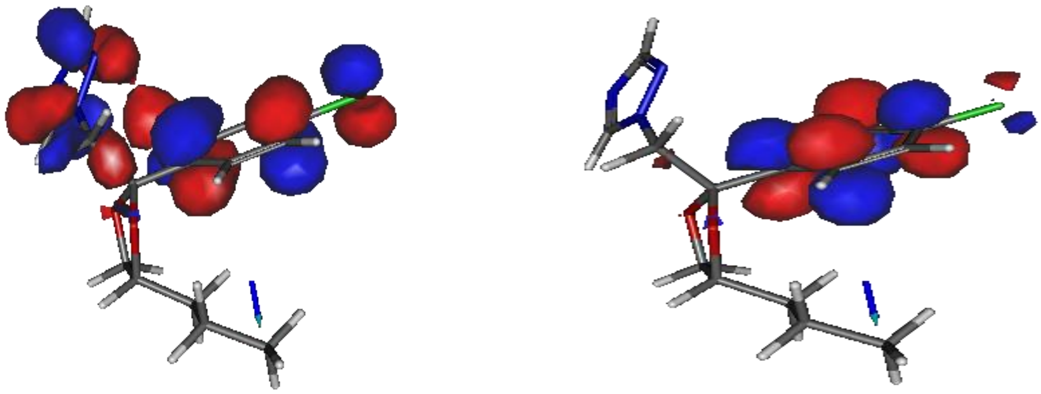

Fenbuconazole

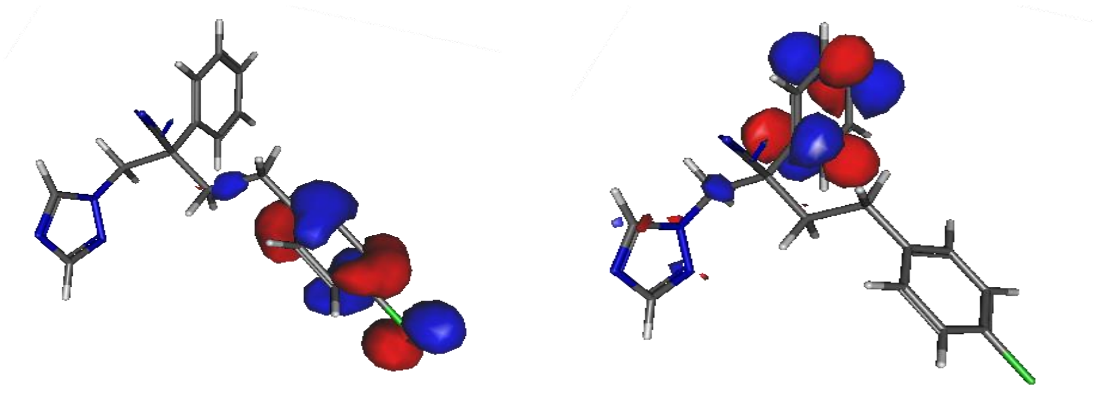

Flutriafol

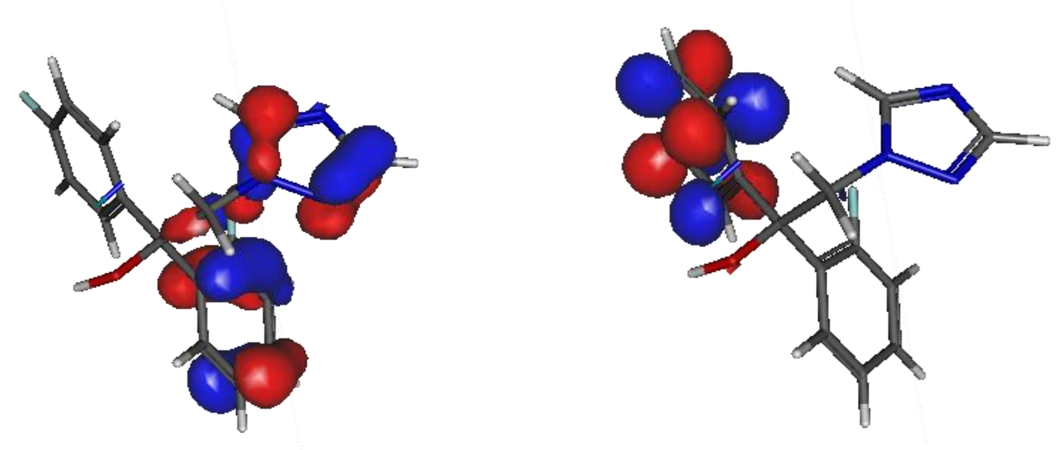

Triadimefon

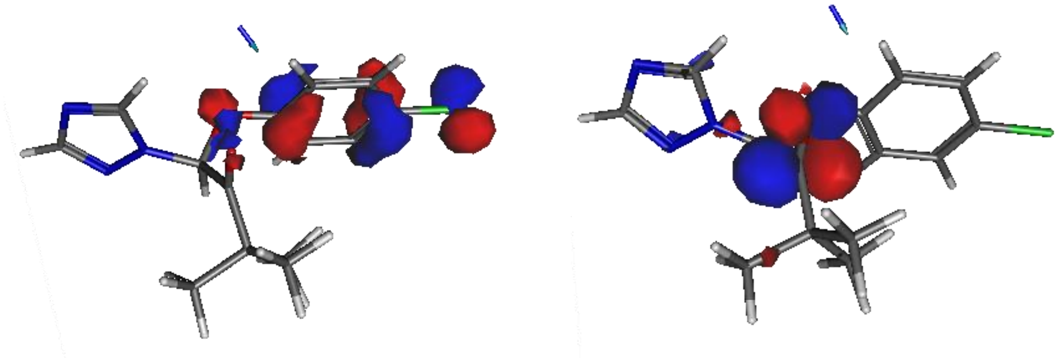

Carfentrazone-ethyl

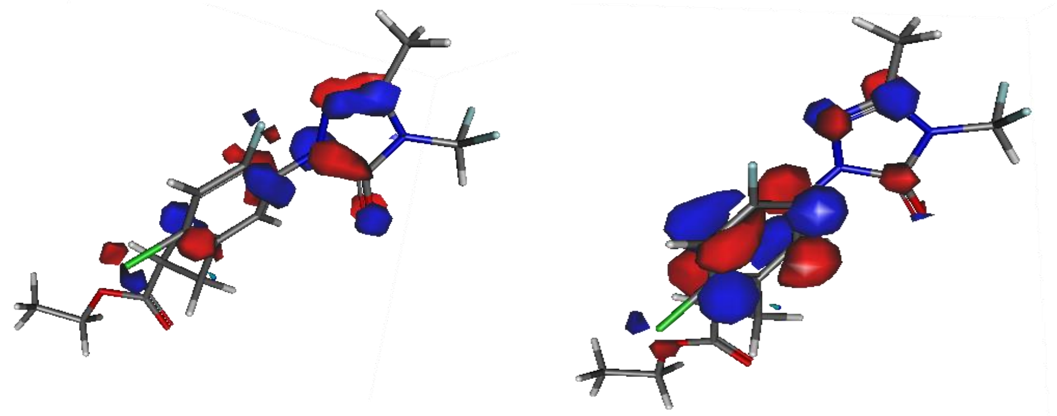

Figure S2: Representations of HOMO and LUMO isosurfaces for the antagonist triazoles computed using the single point at B3LYP/def2-SV(P) method on the structure obtained following complete optimisation with PM7.

.

HOMONL **HOMO** **LUMO**

Fadrozole

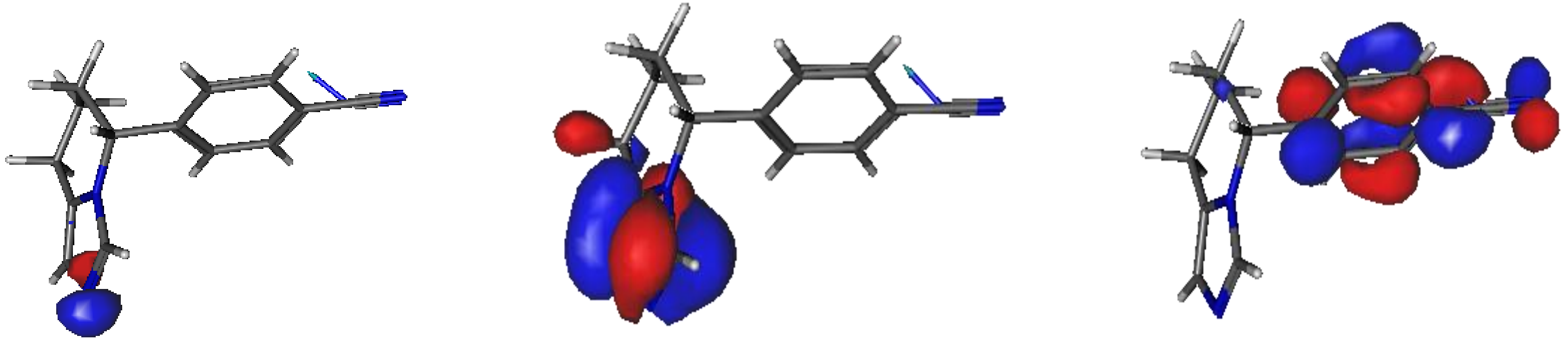

Triflumizole

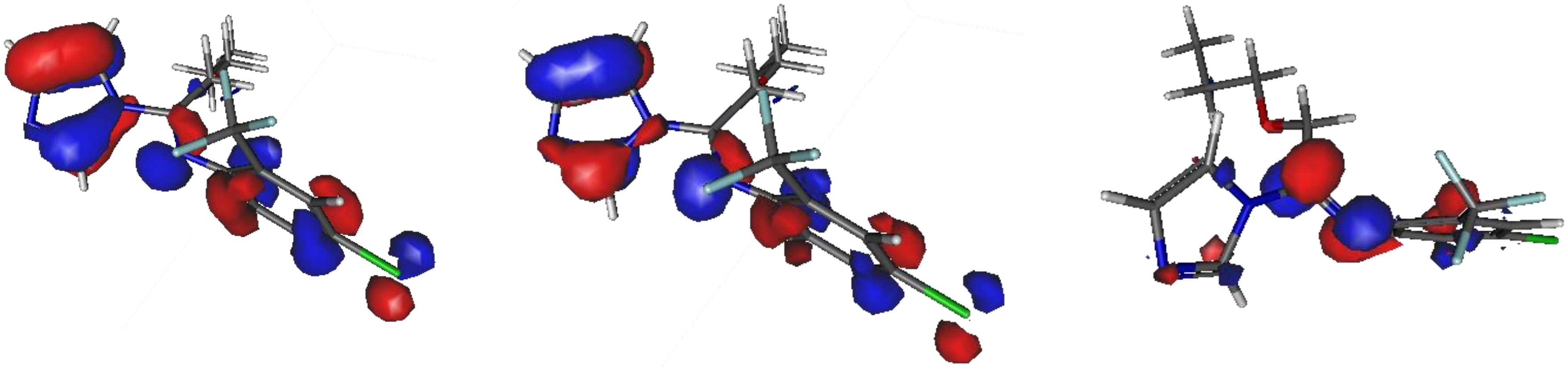

6-Thioguanine

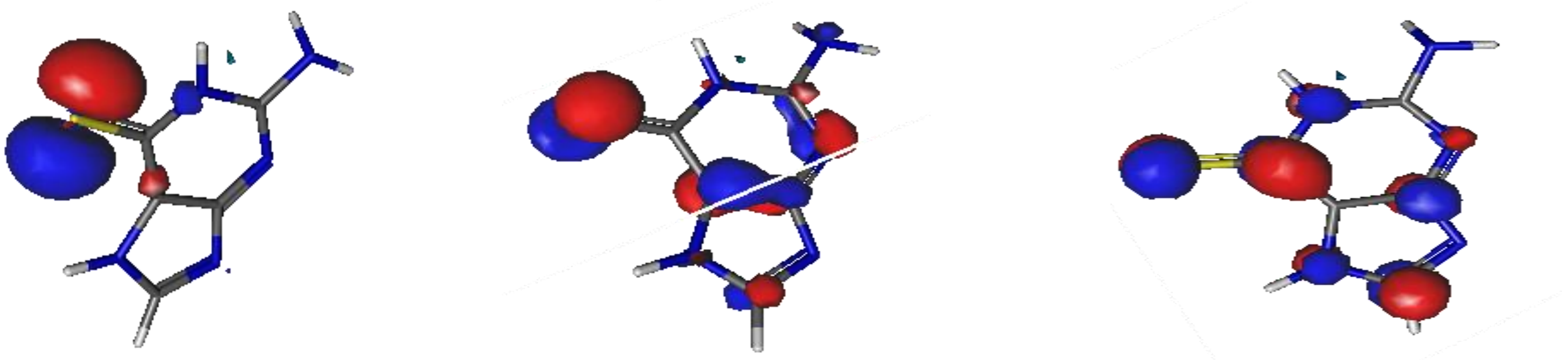

Liarozole

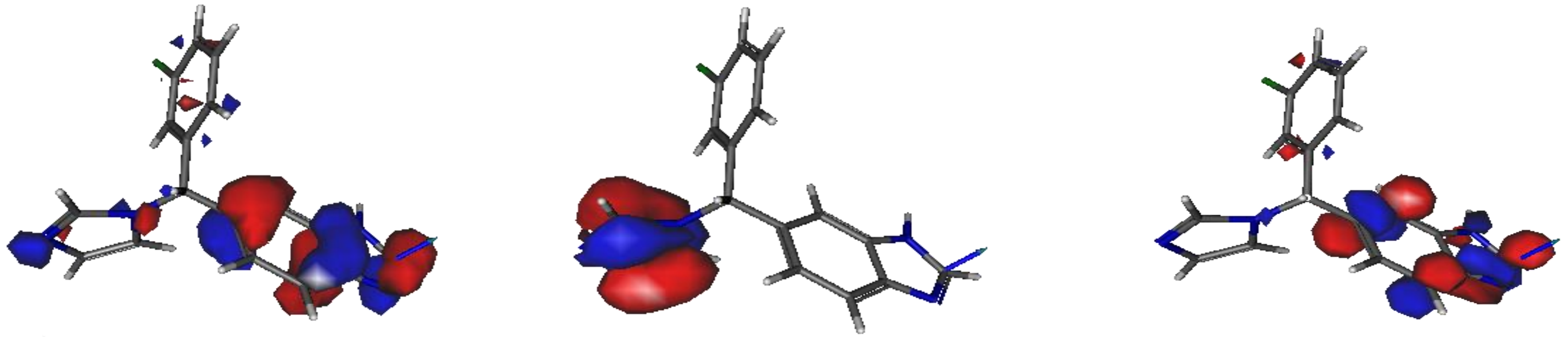

Imazalil

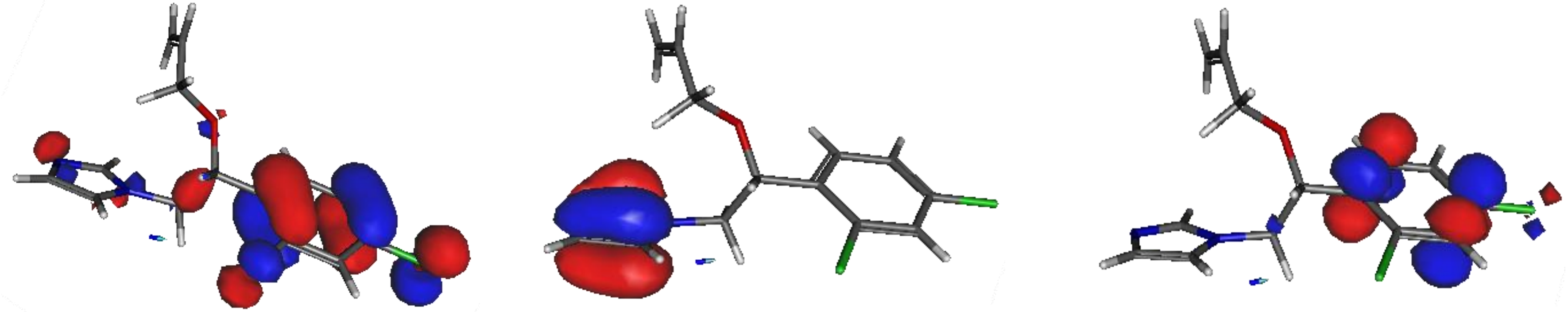

Bifonazole

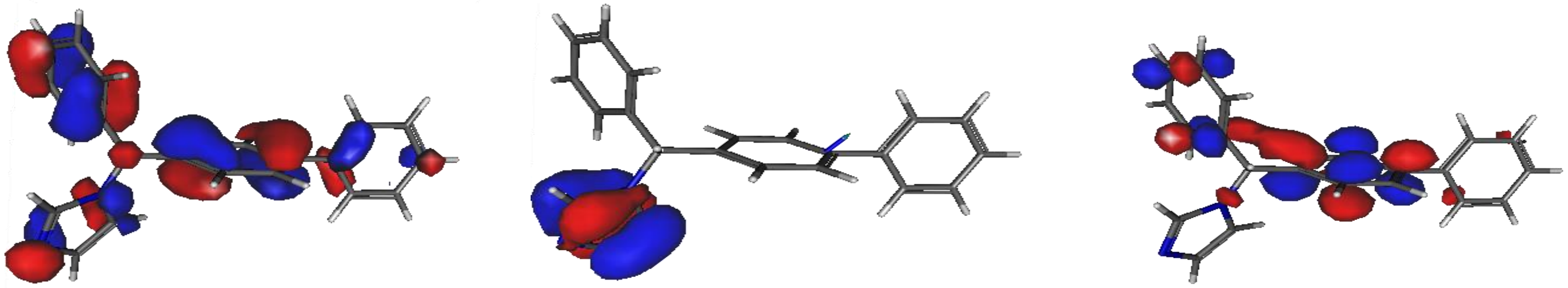

Luliconazole

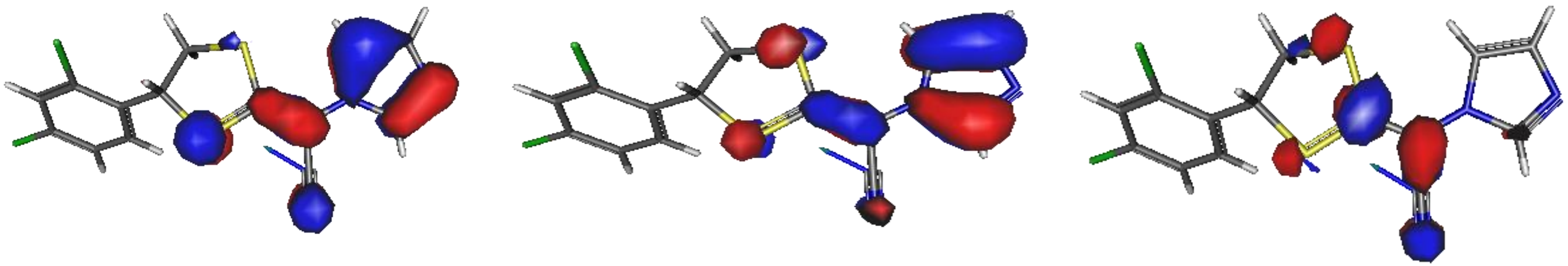

Prochloraz

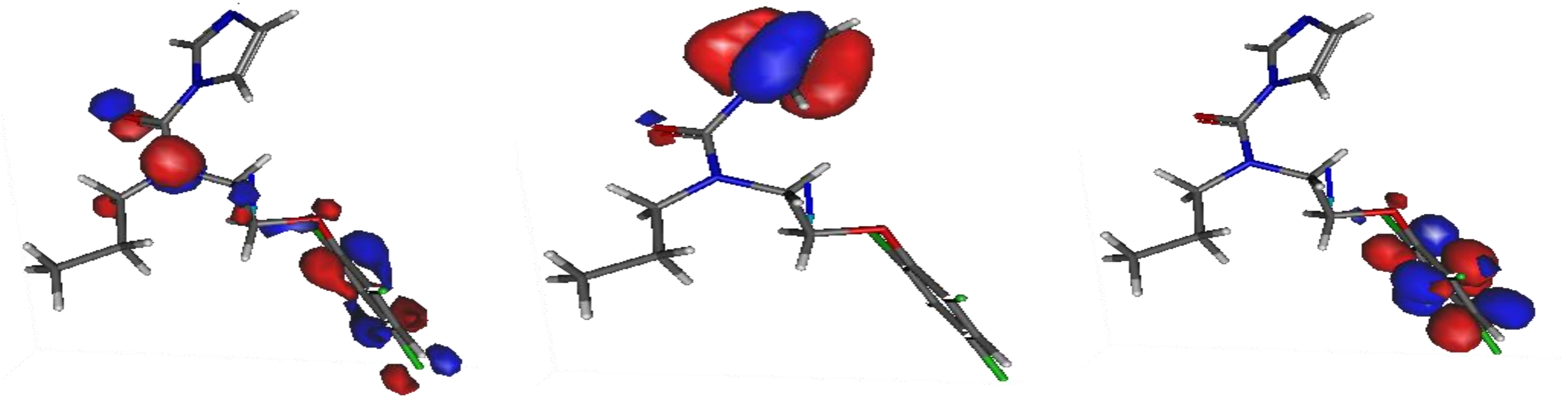

Clofarabine

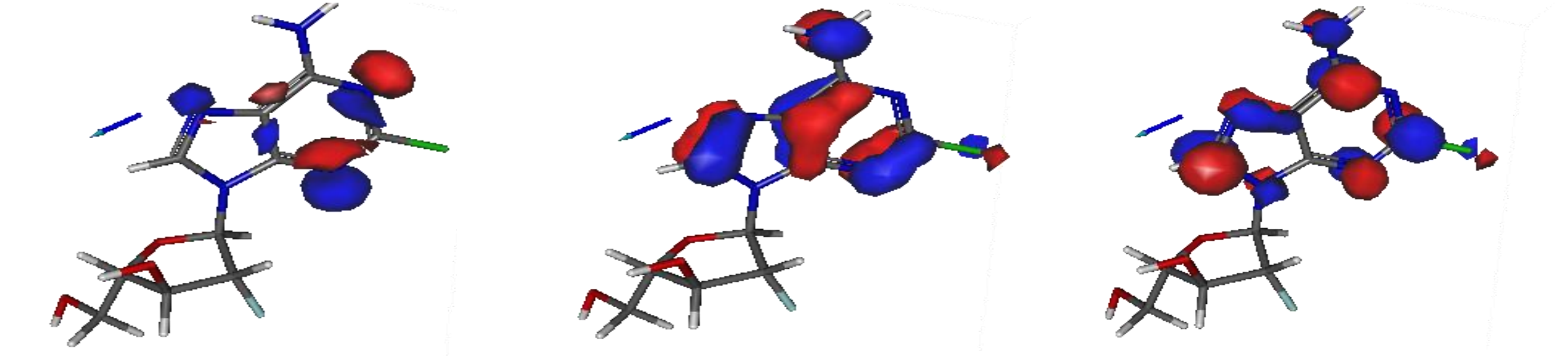

Oxiconazole

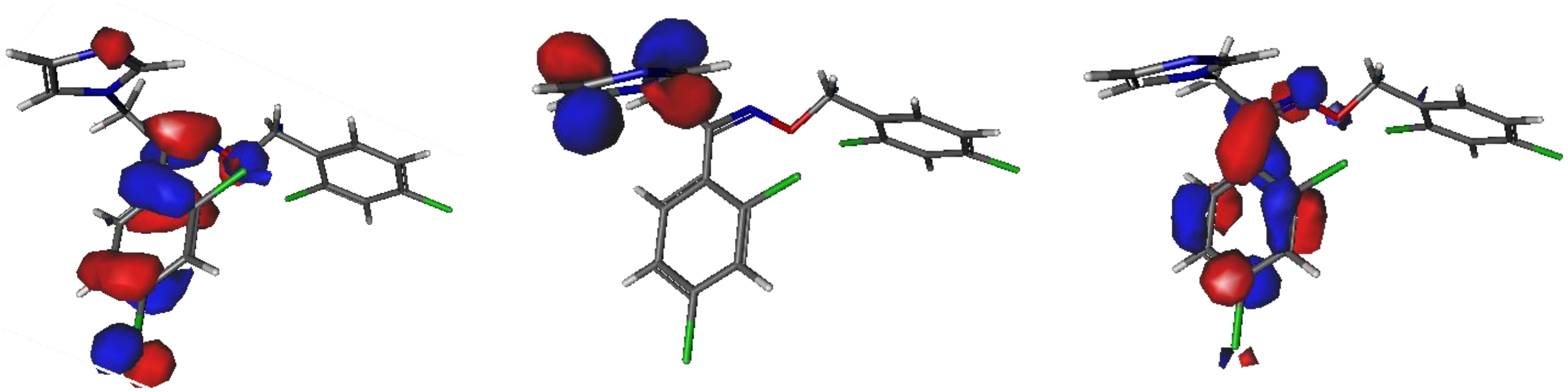

Sulconazole

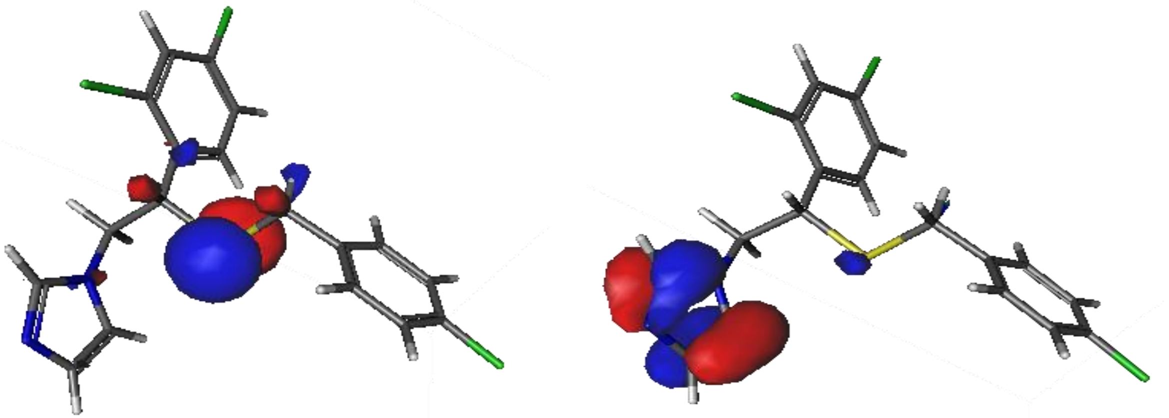

Lanoconazole

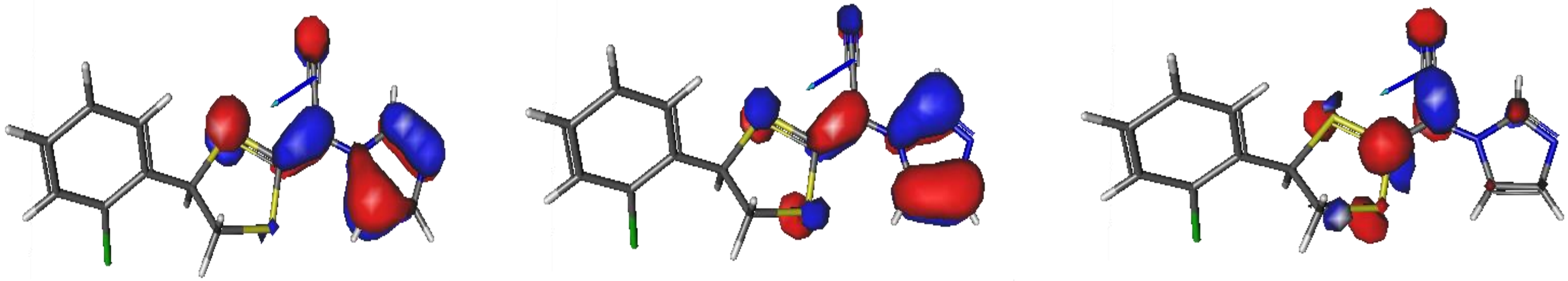

Sertaconazol

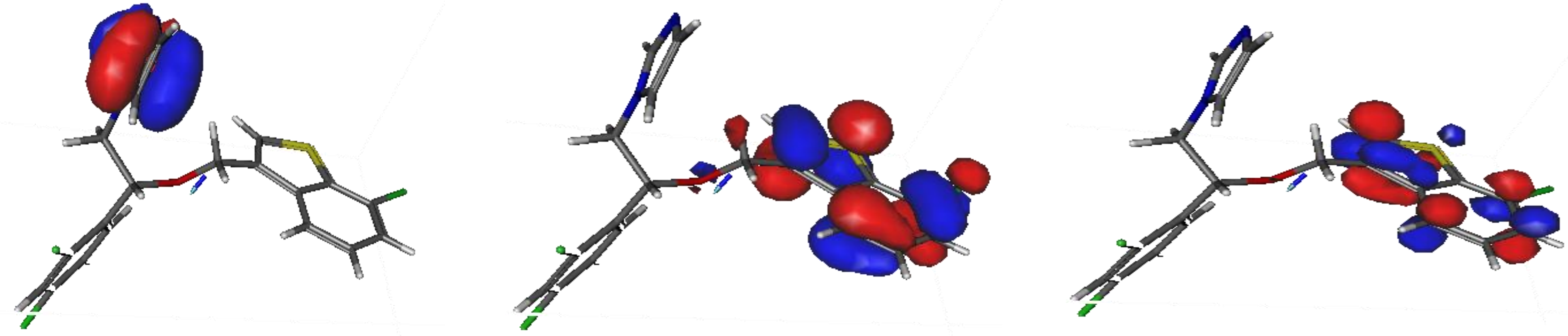

Tioconazole

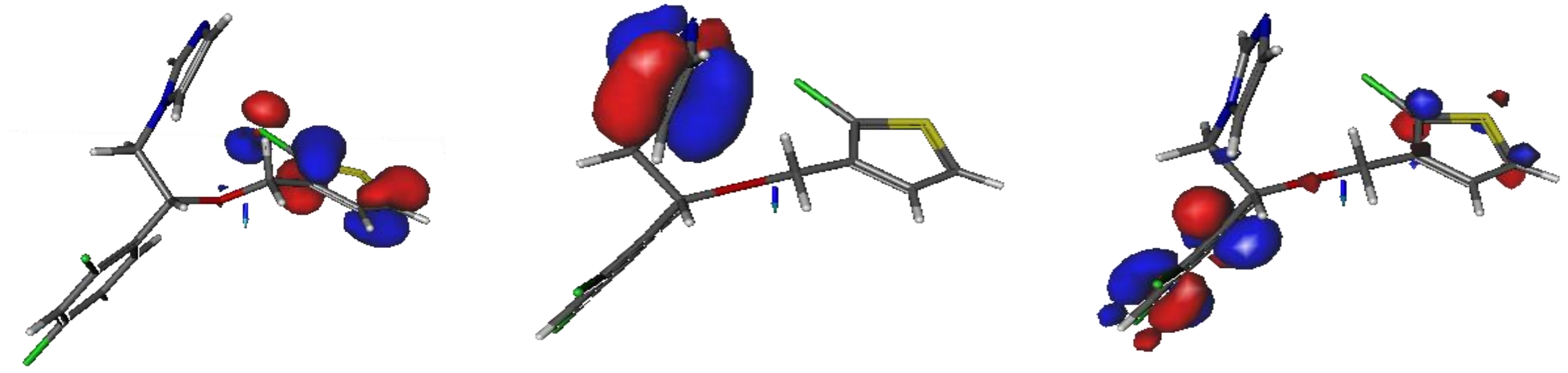

Metrifudil

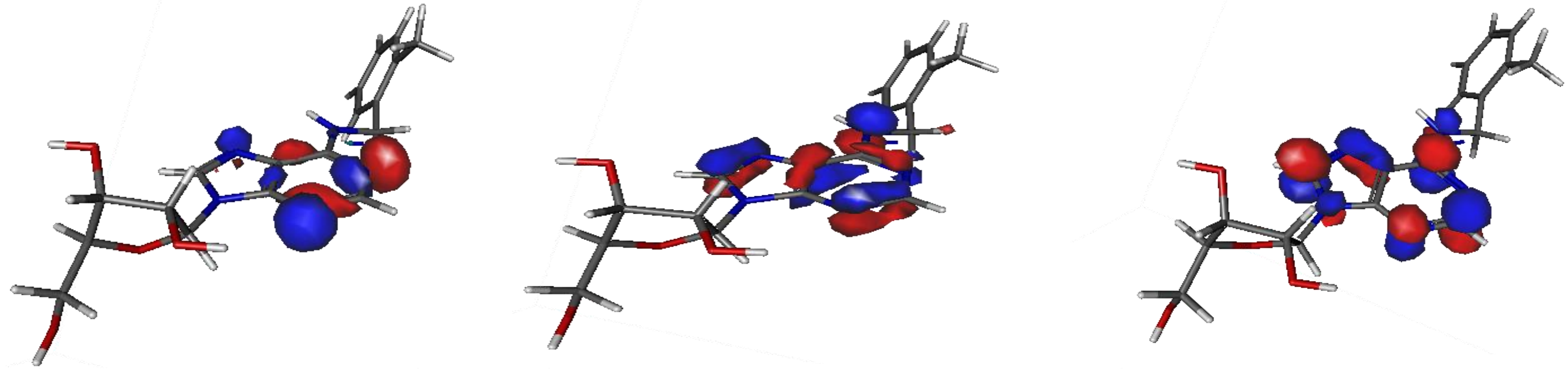

1-[2-(Trifluoromethyl)phenyl]-1H-imidazole

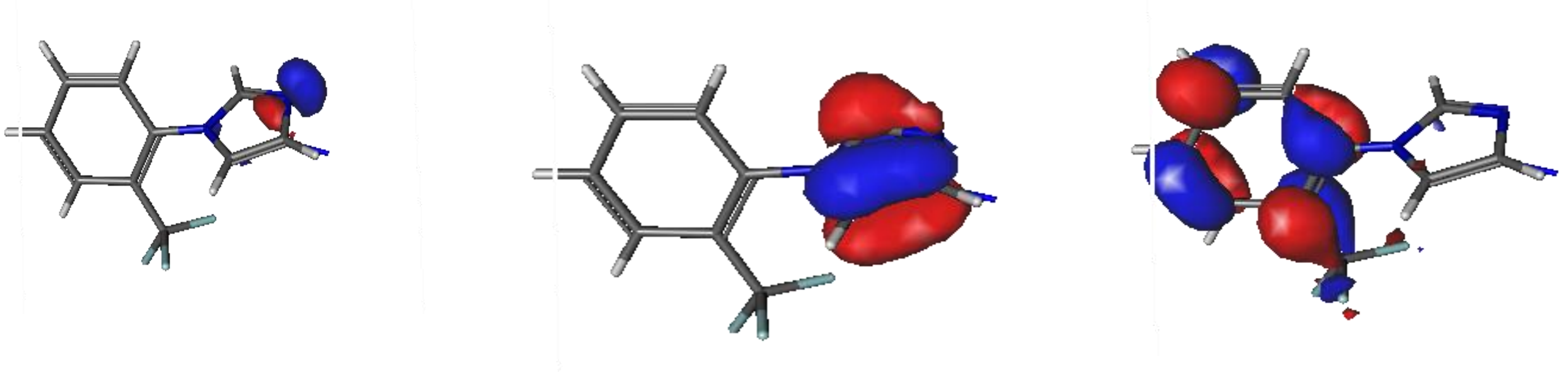

Imazodan

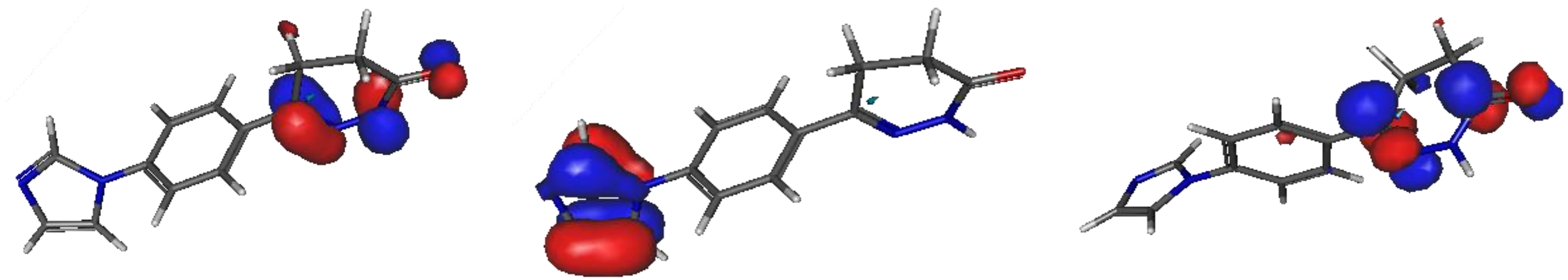

Miconazole

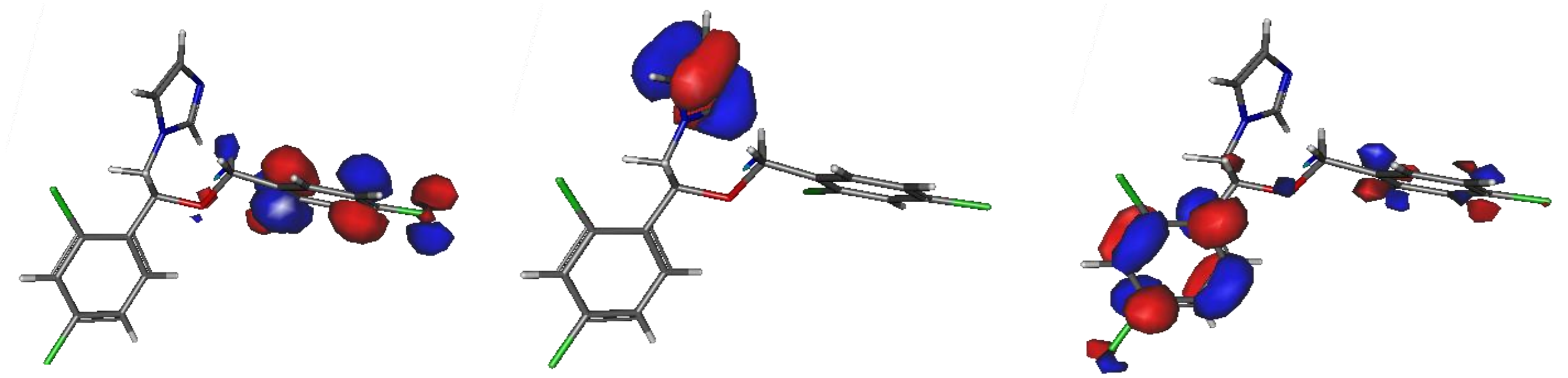

Isoconazole

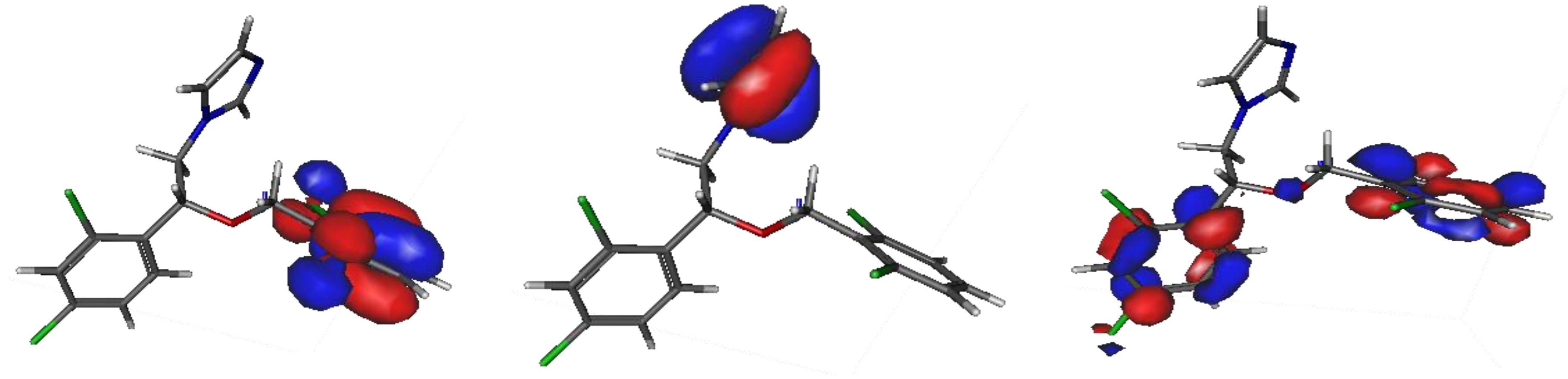

Climbazole

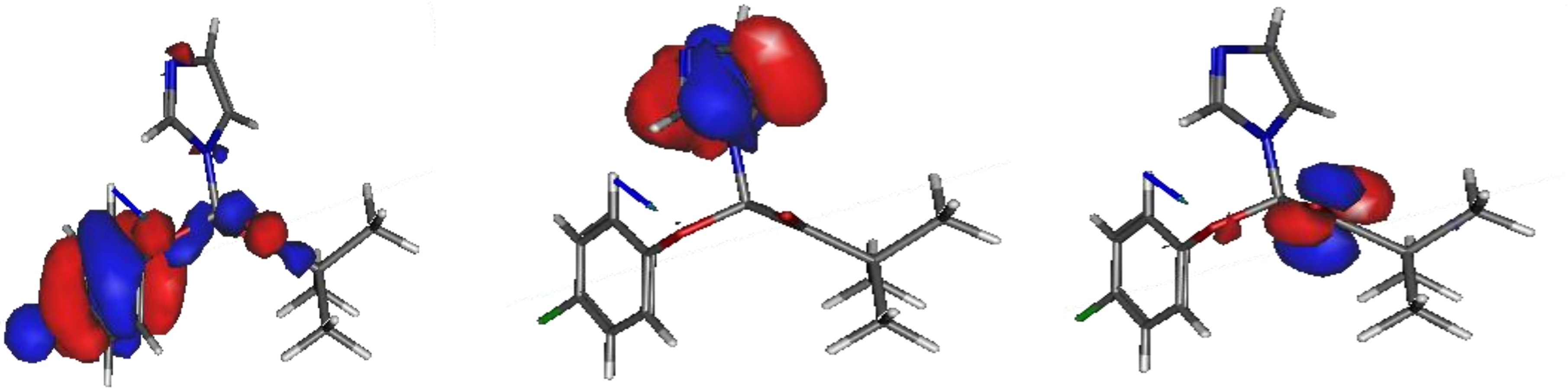

Butoconazole

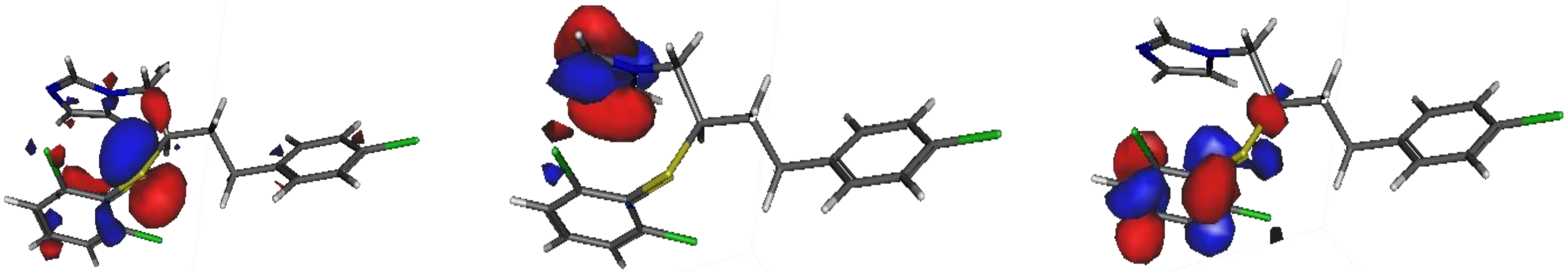

Timiperone

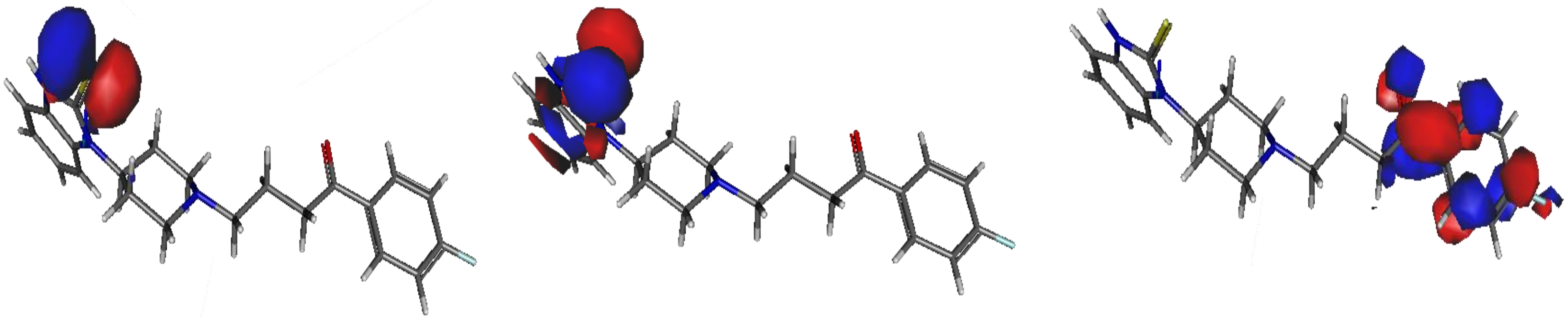

Triclabendazole

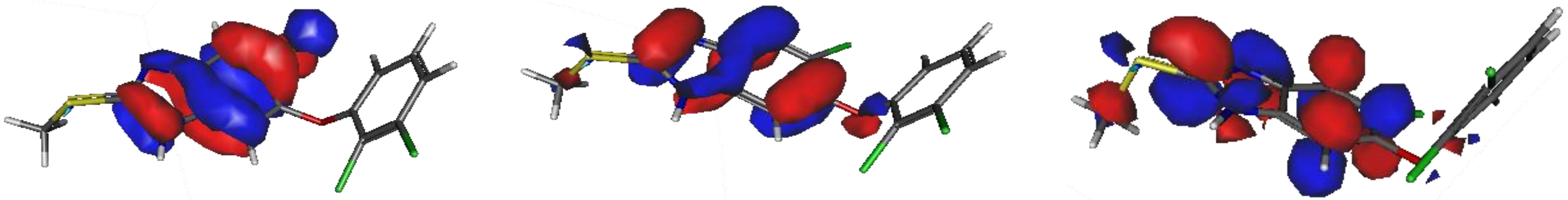

Medetomidine

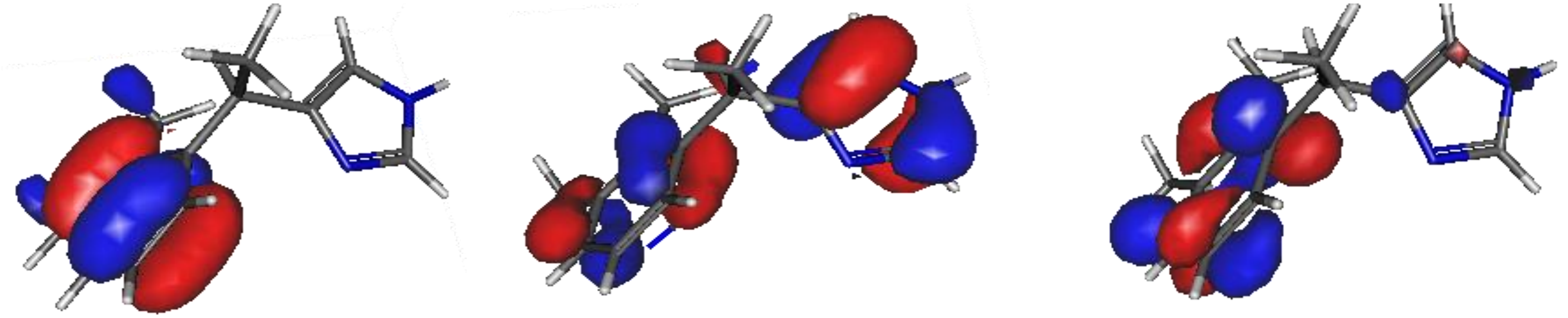

Zaldaride

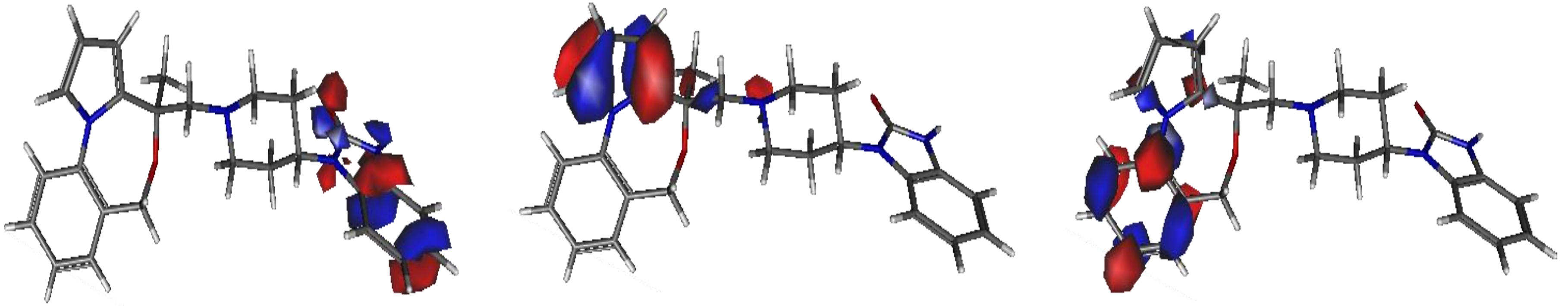

CJ-013610

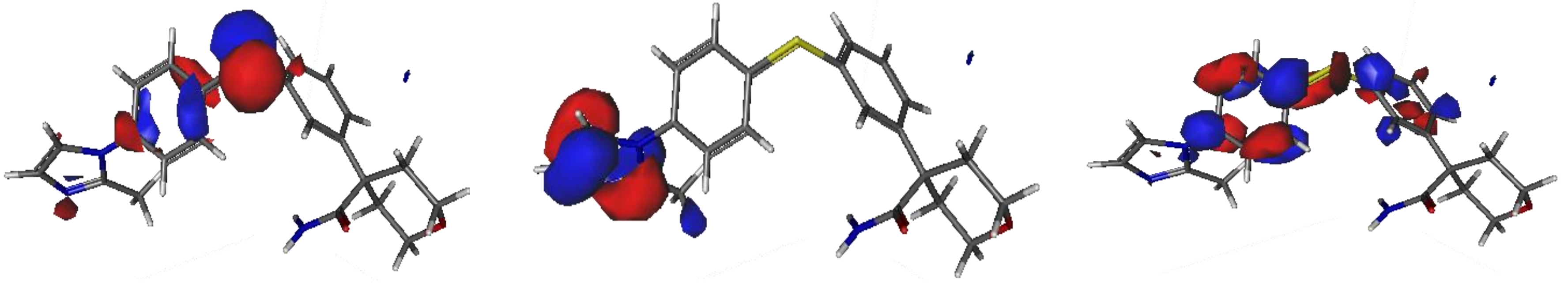

Surinabant

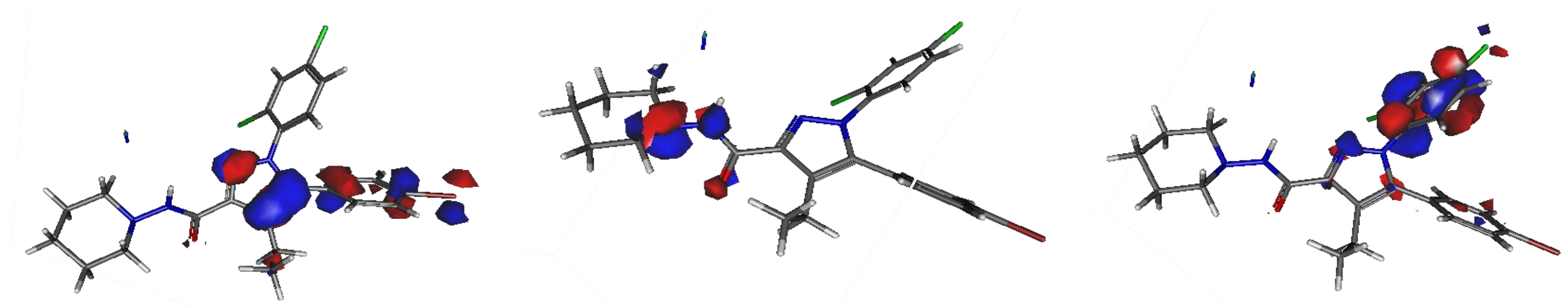

Figure S3: Representation of HOMO-1 (HOMONL), HOMO and LUMO isosurfaces of antagonist diazoles computed using the single point at B3LYP/def2-TZVP method on the structure obtained following complete optimisation with PM7

Figure S4

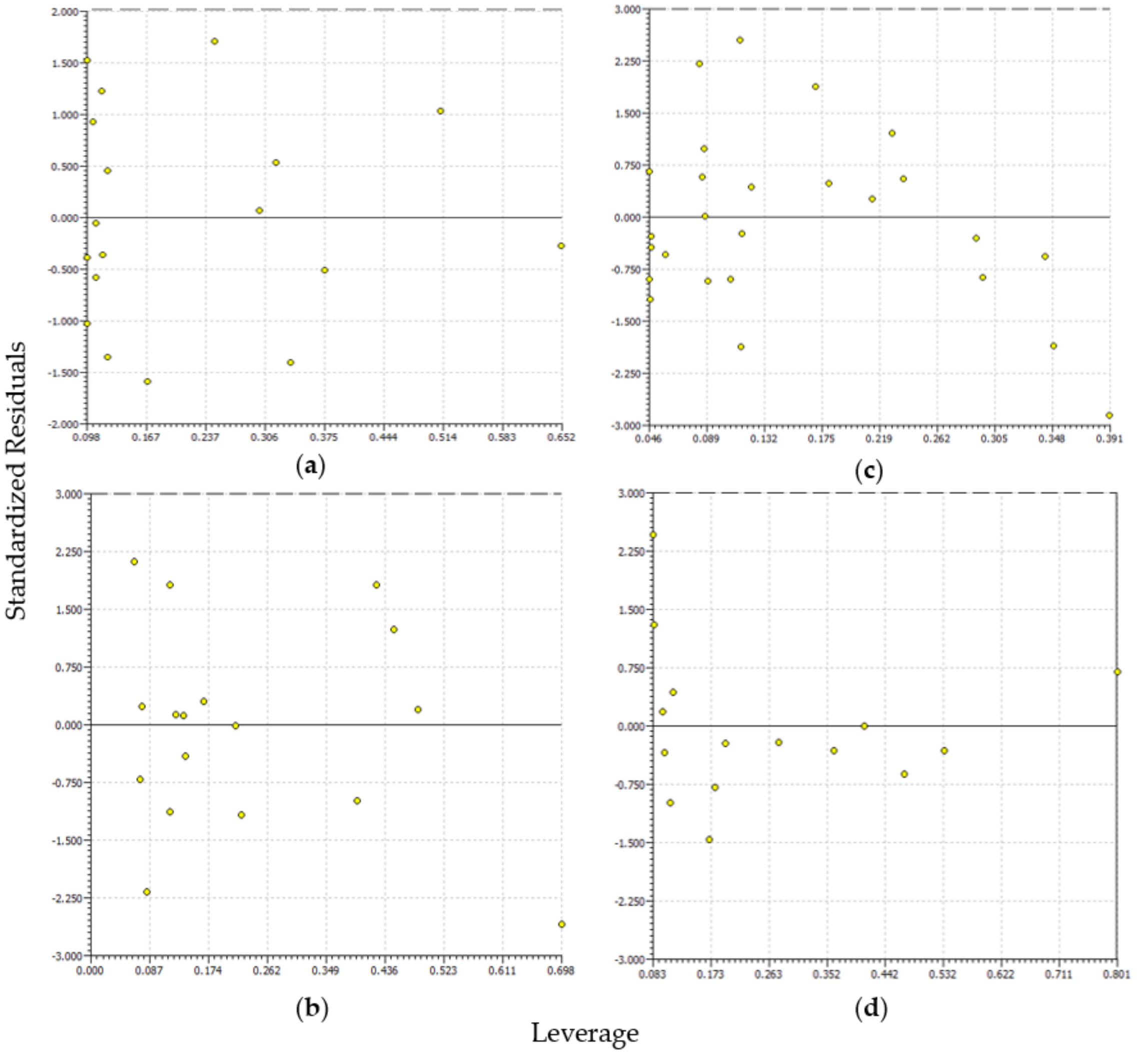

Figure S4. Williams plots of leverage vs standardised residuals for equations (1, 2, 4, 5) for: (**a**) agonist monazoles *(Thiazole/oxazole)*; (**b**) agonist Diazoles *(imidazoles and benzimidazole);* (**c**) antagonist Diazoles (*imidazoles and benzimidazole);* (**d**) antagonist Triazoles.
